# A heritable profile of six miRNAs in autistic patients and mouse models

**DOI:** 10.1038/s41598-020-65847-8

**Published:** 2020-06-09

**Authors:** Yusuf Ozkul, Serpil Taheri, Kezban Korkmaz Bayram, Elif Funda Sener, Ecmel Mehmetbeyoglu, Didem Behice Öztop, Fatma Aybuga, Esra Tufan, Arslan Bayram, Nazan Dolu, Gokmen Zararsiz, Leila Kianmehr, Feyzullah Beyaz, Züleyha Doganyigit, François Cuzin, Minoo Rassoulzadegan

**Affiliations:** 1 Erciyes University Medical Faculty, Medical Genetics Department, Kayseri, Turkey; 2 Erciyes University, Betul-Ziya Eren Genome and Stem Cell Center, Kayseri, Turkey; 3 Erciyes University Medical Faculty, Medical Biology Department, Kayseri, Turkey; 4 Ankara University, Medical Faculty, Child and Adolescent Psychiatry Department, Ankara, Turkey; 5 Baskent University, Medical Faculty, Physiology Department, Ankara, Turkey; 6 Erciyes University Veterinary Faculty, Histology and Embryology Department, Kayseri, Turkey; 7 Bozok University, Medical Faculty, Histology and Embryology Department, Yozgat, Turkey; 8 Université Côte d’Azur, CNRS, Inserm, France

**Keywords:** Neurodevelopmental disorders, Transgenic organisms

## Abstract

Autism spectrum disorder (ASD) is a group of developmental pathologies that impair social communication and cause repetitive behaviors. The suggested roles of noncoding RNAs in pathology led us to perform a comparative analysis of the microRNAs expressed in the serum of human ASD patients. The analysis of a cohort of 45 children with ASD revealed that six microRNAs (miR-19a-3p, miR-361-5p, miR-3613-3p, miR-150-5p, miR-126-3p, and miR-499a-5p) were expressed at low to very low levels compared to those in healthy controls. A similar but less pronounced decrease was registered in the clinically unaffected parents of the sick children and in their siblings but never in any genetically unrelated control. Results consistent with these observations were obtained in the blood, hypothalamus and sperm of two of the established mouse models of ASD: valproic acid-treated animals and *Cc2d1a*^+/−^ heterozygotes. In both instances, the same characteristic miRNA profile was evidenced in the affected individuals and inherited together with disease symptoms in the progeny of crosses with healthy animals. The consistent association of these genetic regulatory changes with the disease provides a starting point for evaluating the changes in the activity of the target genes and, thus, the underlying mechanism(s). From the applied societal and medical perspectives, once properly confirmed in large cohorts, these observations provide tools for the very early identification of affected children and progenitors.

## Introduction

Autism spectrum disorders (ASDs) encompass a range of disorders characterized by impaired social interactions and communications, together with repetitive stereotypic behaviors (refs. ^1–5^ for recent reviews). The genetic architecture underlying the range of ASD symptoms has been investigated (reviewed by Iakoucheva *et al*.^1^). Mutations in more than 100 genes involved in brain development and neural activity have been identified in patients and are thought to confer a risk for ASD^2,3^, but a constant association that would suggest a causal relationship has not been observed. The same conclusion was recently reached from a large-scale exon sequencing analysis^4^. Hence, mouse models that reproduce characteristic elements of the disease have been developed^5^. As in other instances, attention has recently been focused on a peculiar class of regulatory alterations that modifies noncoding (nc) RNAs^6^ with putative regulatory functions in the synthesis of proteins. One class of these alterations comprises the genes encoding 22 nt-long RNA (often abbreviated miRNAs) that regulate the expression of homologous target genes by blocking translation and inducing the degradation of the mRNAs^7^. Among the miRNA genes in the mammalian genome (several hundred in the human genome), a large subset is expressed in the brain^8^, and dysfunctions of particular miRNAs have been tentatively associated with neuropathological conditions, including ASD^9,10^, with however diverging patterns of expression. They may reflect still unknown complexities of the disease itself but they may also be the result of different analytical protocols.

Here, we report a characteristic miRNA profile of expression of six miRNA genes detected by quantitative qRT-PCR analysis in human patients and further evidenced in two established animal models of the disease. MicroRNAs miR-19a-3p, miR-361-5p, miR-3613-3p, miR-150-5p, miR-126-3p, and miR-499a-5p were found to be expressed at low to very low levels in the serum of 45 human patients with autism. The clinically healthy progenitors and siblings of the patients showed levels of these microRNAs intermediate between those of controls and the reduced expression of patients. The same pattern was observed in two mouse models, one generated via the injection of valproic acid (VPA), a drug known to induce autism in humans^11–13^ and rodents^14,15^ and another one resulting from heterozygosity of the *Cc2d1a*^+/−^ locus, a gene of the ASD constellation encoding a transcriptional repressor of serotonin receptors^16,17^. Among more than one-hundred gene reported to exhibit an altered expression pattern in human ASD instances, mice with a *Cc2d1a* mutation were chosen because, the loss of the gene affects serotonin receptors involved in the normal and pathological brain development^18–20^ and mutants were considered as valuable models of ASD. The same abnormal, disease-associated profile of expression of the same six microRNAs genes, in 45 patients, from multiplex (more than one child with autism) and simplex (one child with autism) families compared with their families and controls further extended to two of the established animal models.

## Results

### Altered serum miRNA profiles in a cohort of 45 autistic patients

A cohort of patients with autism, unaffected family members and healthy controls was assembled at the Erciyes University School of Medicine Hospital, Kayseri, Turkey (n = 189) (clinical evaluation results are presented in Supplementary Table 1a). It comprised affected children (n = 45, 2–13 years old 31 boys and 14 girls), their unaffected siblings (n = 33, 1–20 years old, 17 boys and 16 girls), their parents (n = 74, mothers 23–45 years old, fathers 24–51 years old), control age-matched children (n = 21, 3–16 years, 10 boys and 11 girls) and 16 control parents, among families 9 multiplex (more than one child with autism) and 28 simplex (one child with autism).

The serum miRNA expression profiles of all the family members (mothers, fathers, sisters, brothers and the autistic children) were compared to those of the age- and sex-matched healthy controls. MicroRNA analysis was carried out by qRT-PCR analysis of the serum samples of a total of 189 participants. The complete raw qRT-PCR data are provided in Supplementary Table 1b, and the technique is described in the Methods section. Statistically significant results (p < 0.05) were registered for 280 miRNAs in the children with autism and their families compared to the controls.

**Figure 1:**
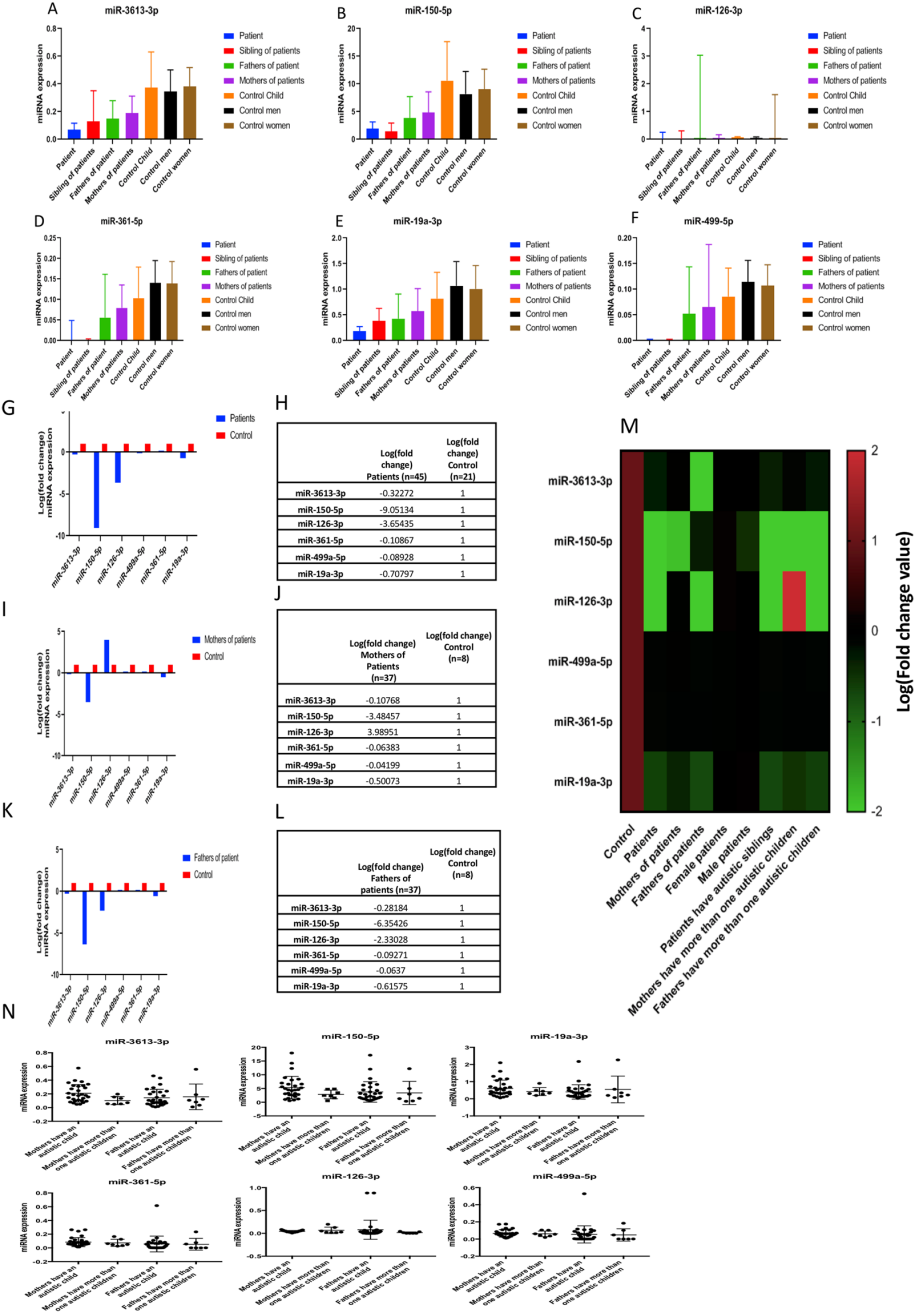
Decreased expression of the six-miRNAs miR-3613-3p, miR-150-5p, miR-126-3p, miR-361-5p, miR-19a-3p, and miR-499a-5p in children with autism and their fathers and mothers compared to age- and sex-matched healthy controls. In the cohort of patients with autism assembled in Kayseri, Turkey, we performed qRT-PCR as described in the Methods to analyze the microRNA profiles of serum samples from 45 autistic children (2–13 years old, 31 boys and 14 girls), their 33 siblings (1–20 years old, 17 boys and 16 girls), their 74 parents (mothers 23–45 years old, fathers 24–51 years old) and 21 control age-matched healthy children (3–16 years old, 10 boys and 11 girls) and their 16 parents, for a total of 189 participants. The expression levels of six-miRNAs miR-3613-3p (**A**), miR-150-5p (**B**), miR-126-3p (**C**), miR-361-5p (**D**), miR-19a-3p (**E**), and miR-499a-5p (**F**) showed statistically significant differences (p < 0.05). The six-miRNA-transcript profiles graphed for a multiplex family (with more than one child with autism), and the tables below show the log fold-change rates for children with autism to control children (**G**, **H**) and their mothers (**I**,**J**) and fathers to father control (**K**,**L**) compared to age- and sex-matched healthy controls (p < 0.0001). Heatmap shows the fold-change variation according to color (**M**), and in the figure, the columns represent the groups, and the rows represent the miRNAs (red, black and green correspond to upregulated, unchanged and downregulated, respectively). N. shows 6 miRNA expression profile in multiplex (more than one child with autism, n = 9) and simplex (one child with autism, n = 28) families.

While most of the microRNAs tested (raw results listed in Supplementary Table 1c), exhibited normal (taken as 100 percent) to lower levels in the patients expression levels in the range of 1 to 10 percent of the healthy control level were noted for six of them in Fig. 1 and Table 1: miR-3613-3p (Fig. 1A), miR-150-5p (Fig. 1B), miR-126-3p (Fig. 1C), miR-361-5p (Fig. 1D), miR-19a-3p (Fig. 1E), miR-499a-5p (Fig. 1F) and in Fig. 1, Table 1, Supplementary Tables 1 and Supplementary Fig. 1. In Fig. 1G–L are shown successively patients to control (Fig. 1G,H), mothers of patients to controls (Fig. 1I,J) and fathers of patients to controls (Fig. 1K,L), patients and relatives with lower levels to controls groups. Table 2 shows fold change (numeric values) and Fig. 1M shows the fold change values of the six-miRNAs differentially expressed in the serum of the different groups indicated by color. Figure 1N shows nine multiplex (more than one child with autism) to twenty-eight simplex (one child with autism) families. No difference between two groups (multiplex/simplex) in the levels of the six miRNAs were observed.

**Table 1:** Transcription values in different groups for “Six-miRNAs”.

	miR-3613-3p	miR-150-5p	miR-126-3p	miR-361-5p	miR-499a-5p	miR-19a3p
Patients	0.069 ± 0.05	1.900 ± 1.22	0.001 ± 0.25	0.001 ± 0.05	0.001 ± 0.002	0.182 ± 0.09
Sibling of Patients	0.130 ± 0.22	1.458 ± 1.49	0.002 ± 0.30	0.002 ± 0.002	0.001 ± 0.002	0.383 ± 0.24
Father of Patients	0.150 ± 0.15	3.864 ± 3.84	0.044 ± 2.99	0.056 ± 0.11	0.053 ± 0.09	0.428 ± 0.48
Mother of Patients	0.190 ± 0.19	4.844 ± 3.74	0.046 ± 0.12	0.080 ± 0.06	0.065 ± 0.12	0.574 ± 0.43
Child Controls	0.373 ± 0.37	10.570 ± 7.06	0.066 ± 0.03	0.103 ± 0.08	0.086 ± 0.05	0.817 ± 0.51
Father Controls	0.344 ± 0.34	8.196 ± 4.06	0.058 ± 0.03	0.140 ± 0.05	0.114 ± 0.04	1.069 ± 0.47
Mother Controls	0.380 ± 0.38	9.062 ± 3.6	0.055 ± 1.56	0.139 ± 0.05	0.107 ± 0.04	1.009 ± 0.45

Transcription profiles of “Six-miRNAs” (raw data after normalization) in children with autism and their siblings, mothers and fathers compared to age- and sex-matched healthy controls (p < 0.0001) (Mann-Whitney tests).

**Table 2:** Decreased expression level of the six-miRNAs.

miRNAs	Log(fold change) Control	Log(fold change) Patients (n = 45)	Log(fold change) Mothers of patients (n = 37)	Log(fold change) Fathers of patients (n = 37)	Log(fold change) Female patients (n = 14)	Log(fold change) Male patients (n = 31)	Log(fold change) Patients have autistic siblings (n = 18)	Log(fold change) Mothers have more than one autistic children (n = 9)
miR-3613-3p	1	−0.30436	−0.03851	−6.806063758	−0.00677	−0.0195	−0.32272	−0.10768
miR-150-5p	1	−8.68965	−1.89998	−0.313461259	0.092496	−0.47326	−9.05134	−3.48457
miR-126-3p	1	−3.46494	−0.02768	−2.1599325	0.095035	−0.00107	−3.65435	3.989511
miR499a-5p	1	−0.08505	−0.03757	−0.091225794	0.000171	−0.00114	−0.10867	−0.06383
miR-361-5p	1	−0.102	−0.05434	−0.066004089	0.000126	−0.00084	−0.08928	−0.04199
miR-19a-3p	1	−0,63541	−036743	−0,71661666	−0,01343	−0,042042	−0,70797	−0,50073

The log fold change rates of six differentially expressed miRNAs in the serum of the different groups.

### Intermediate profiles of apparently healthy relatives

Intermediate levels were recorded for the same six-miRNAs in the serum of the healthy siblings, fathers and mothers of the affected children. Although the levels of these miRNAs were higher than in the affected children, they were significantly lower by 40 to 50 percent Fig. 1 and Table 1 (at least one of the parents) compared with those in genetically unrelated controls (Fig. 1, Supplementary Table 1, and Supplementary Fig. 1). All comparisons are separately listed in Supplementary Fig. 1, including those between (a) patient mothers and healthy mothers; (b) patient fathers and healthy fathers; (c) autism patients and healthy siblings; and (d) female autism patients and healthy female siblings.

### Individual examples of families with either three affected children (multiplex) or one affected child (simplex)

**Figure 2:**
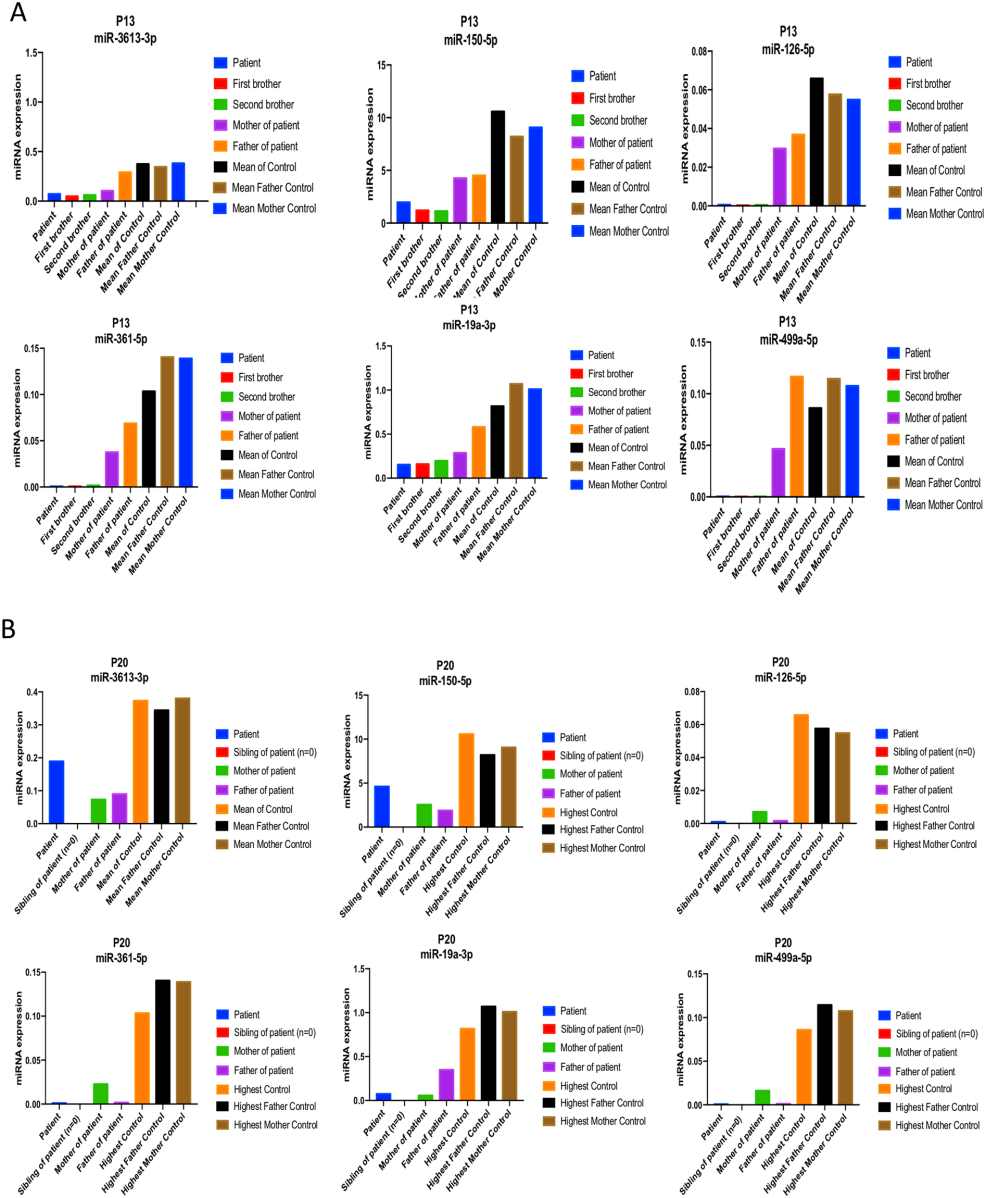
Family profiles for six-miRNAs miR-3613-3p, miR-150-5p, miR-126-3p, miR-361-5p, miR-19a-3p, and miR-499a-5p with a father of three behaviorally affected children. **A**. Family profiles, who has more than one affected child (girl is ASD, one of boys is schizonphrenia), shows six-miRNAs (raw data) compared to mother, and father and the healthy controls. **B**. Family profiles, who has an affected child, shows six-miRNAs compared to the mother, father and healthy controls.

Analyzing the distribution of the markers in large families with ASD and related diseases may be of interest for further studies. In Fig. 2 is shown one example in the present cohort, Fig. 2A illustrates the case of a family with three affected children, including one girl autistic patient born to one mother and two boy patients one with schizophrenia born to a different mother. All six-miRNAs down-regulated in patients presented low levels in the serum blood of daughter patient, her two half -brothers to mother and father (Fig. 2A). Figure 2B in comparison illustrates the case of a family with one affected child with the same results that presents lower level of six-miRNAs to parents and to controls.

### Mouse models of ASD (autism spectrum disorders) confirm the reduced levels of the six microRNAs as characteristic of the disease

**Figure 3:**
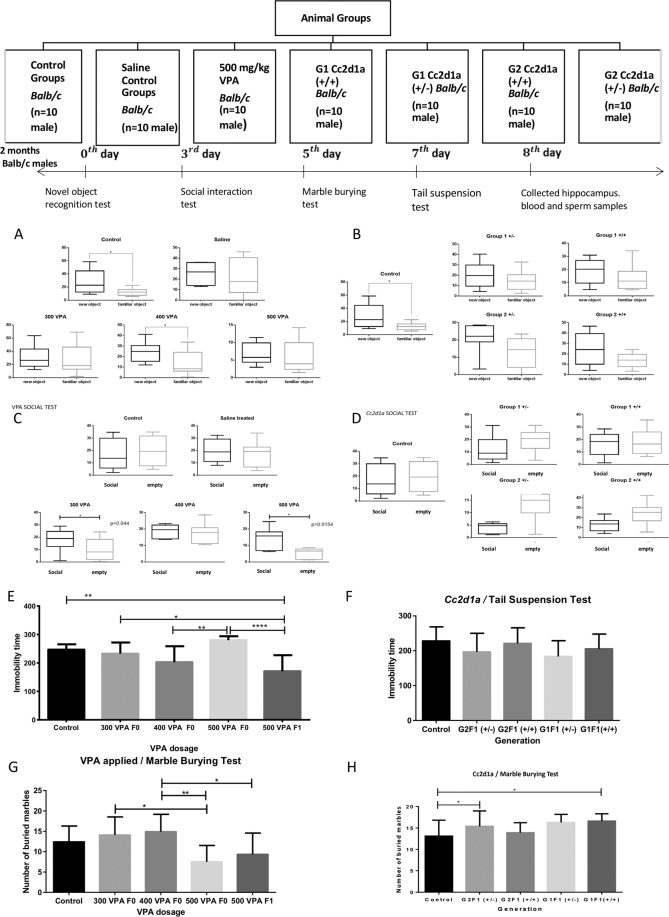
Summary of animals, experiments and timeline. Whole groups (n = 10) adult mice (2 months old, *Balb/c* line) weighting 25-27 g on the test day was conducted behavioral test novel object, social behavioral test, Marble burying test and tail suspension test respectively. *p < 0.05, **p < 0.001, ****p < 0.0001. After behavioral test, whole groups of mice (n = 5) was sacrificed and blood, hippocampus and sperm samples were harvested. VPA (Valproic acid) *Cc2d1a group G1* = *Cc2d1a*^+/−^ *x Cc2d1a*^+/+^*, G2* = *Cc2d1a*^+/−^ *x Cc2d1a*^+/−^, *control* = *Cc2d1a*^+/+^ *x Cc2d1a*^+/+^ crosses are presented. Novel object recognition (NOR) test. Time spent with familiar versus novel objects (see Materials and Methods). Object recognition is measured as the difference in the time spent with the familiar versus the new objects. (**A**) Valproic acid-treated males (500 mg/kg dose). (**B**) *Cc2d1a* group, offspring from *G1* = *Cc2d1a*^+/−^ *x Cc2d1a*^+/+^*, G2* = *Cc2d1a*^+/−^ *x Cc2d1a*^+/−^, *control* = *Cc2d1a*^+/+^ *x Cc2d1a*^+/+^ crosses are presented. Controls for all groups are normal wild type *Cc2d1a*^+/+^ *Balb/c*, that never been crossed with the mutants mice. Social interaction test To determine whether the interactions varied between groups of mice, the mice were placed in the center of a cage divided into three compartments, where chamber A contained another mouse, and chamber B was empty (see Materials and Methods). They were filmed for 5 minutes to calculate the time that they spend close to the empty compartment or to the compartment occupied by another mouse. **(C)**Valproic acid-treated males (500 mg/kg dose). (**D**) *Cc2d1a* group males, offspring from *G1* = *Cc2d1a*^+/−^ *x Cc2d1a*^+/+^*, G2* = *Cc2d1a*^+/−^ *x Cc2d1a*^+/−^, and *control* = *Cc2d1a*^+/+^ *x Cc2d1a*^+/+^ crosses are presented. Controls for all groups are normal wild type *Balb/c*, that never been crossed with the mutants mice. Tail suspension test The test consists of short-term suspension of mice by their tail (six minutes here). They were filmed and analyzed for immobility posture (see Materials and Methods). (**E)** Valproic acid-treated males (500 mg/kg dose). (**F**) *CC2d1a* group males, offspring from *G1* = *Cc2d1a*^+/−^ *x Cc2d1a*^+/+^*, G2* = *Cc2d1a*^+/−^ *x Cc2d1a*^+/−^, and *control* = *Cc2d1a*^+/+^ *x Cc2d1a*^+/+^ crosses are presented. Controls for all groups are normal wild type *Cc2d1a*^+/+^*, Balb/c*, that never been crossed with the mutants mice. Glass marble burying test Repetitive action was evaluated via the marble burying test (see Materials and Methods). Twenty glass marbles were placed on the surface of clean bedding, and the number of marbles buried in 30 minutes was scored. (**G)** Valproic acid-treated males (500 mg/kg dose). (**H**) *CC2d1a* group males, offspring from *G1* = *Cc2d1a*^+/−^ *x Cc2d1a*^+/+^*, G2* = *Cc2d1a*^+/−^ *x Cc2d1a*^+/−^, *control* = *Cc2d1a*^+/+^ *x Cc2d1a*^+/+^ crosses are presented. Controls for all groups are normal wild type *Cc2d1a*^+/+^*, Balb/c*, that never been crossed with the mutants mice.

While the number of patients, relatives and controls included in the analysis ensured the statistical validity of the results (p < 0.05), their general significance could be questioned because of the apparent divergence of the conclusions from published data generated in much larger cohorts of patients^21^ and the possibility of geographic and ethnographic differences. As a test of the validity of the conclusions regarding the autistic pathology, we checked whether they could be verified in animal models. For this purpose, we chose two distinct models are presented in Fig. 3: mice treated with valproic acid (VPA) and *Cc2d1a* heterozygotes.

#### Valproic acid intraperitoneal injection

VPA is known to perturb brain formation during early development^22^ and to induce characteristic traits of ASD pathology^23,24^. To establish reference phenotypes to which the experimental results could be compared, we chose to follow the development of the ASD-like phenotype in *B6D2* and *Balb/c* males that had received one intraperitoneal injection of VPA at a concentration of 300–700 mg/kg at two weeks of age^25^ (see Supplementary Table 3). We treated the animals after birth rather than treating pregnant females as in previous studies^23,24^ to avoid the multiple effects on embryonic development generated via the mothers during pregnancy as well as the effects on germ cells. In addition, we observed that in two-week-old males, VPA injection did not induce changes in body weight compared to the control group, and sudden dramatic mortality only occurred at high doses (600–700 mg/kg) in both mouse line. In present study on the males, doses responses show reproducible affected phenotype at 500 mg/kg dosage without sudden mortality. In contrast, the injection of pregnant females often leads to an unpredicted arrest of embryonic development and mortality of the mothers either during pregnancy or after the birth of their progenies.

**Figure 4:**
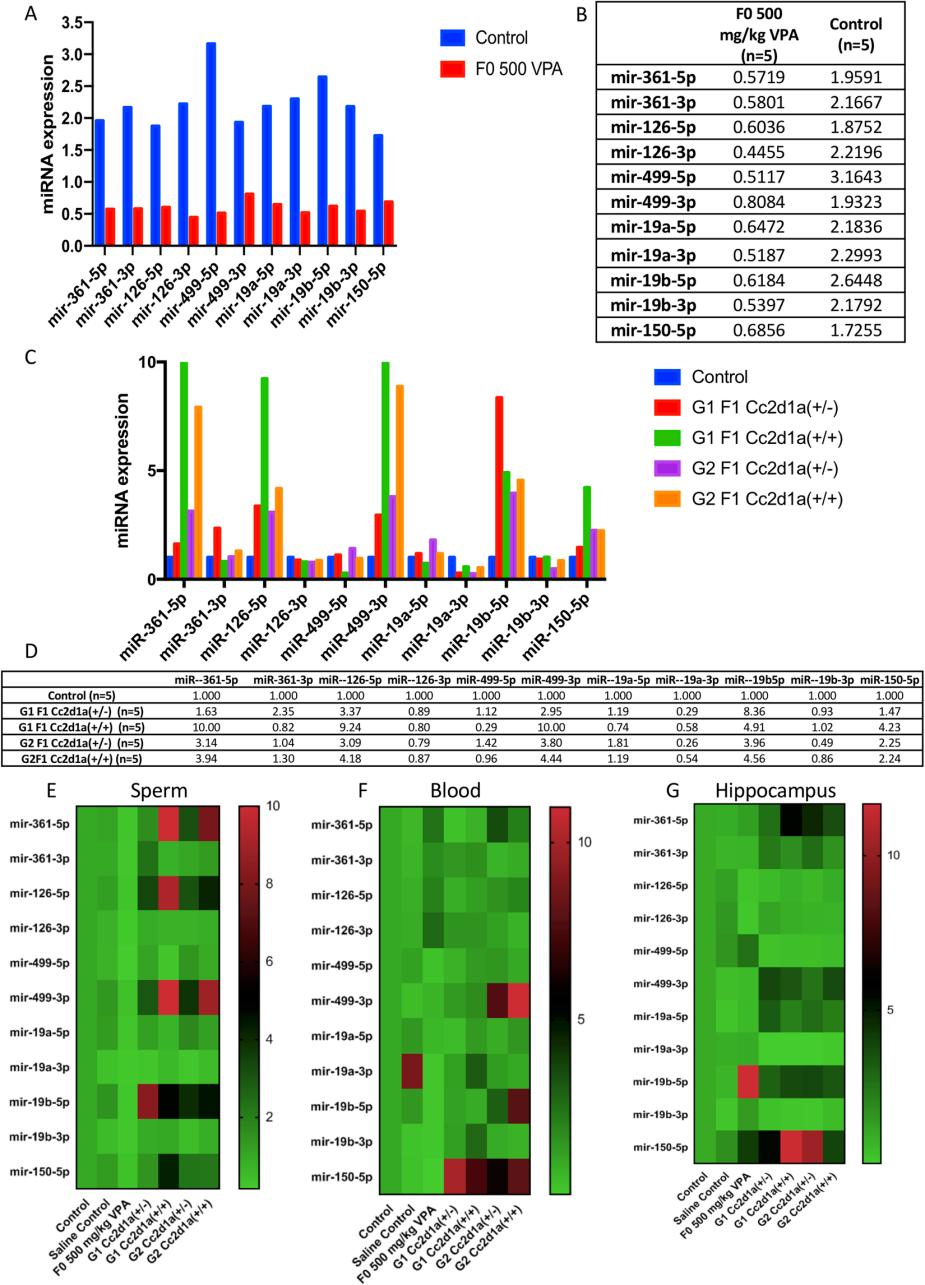
miRNA expression profiles of mouse sperm. Total sperm RNAs from four-month-old *Balb/c* males were tested by q-PCR for microRNAs. A in graph and B in fold change from VPA-treated males (500 mg/kg) are compared to those of controls. C in graph and D in fold change for group and offspring genotypes produced by the following crosses: *G1* = *Cc2d1a*^+/−^ *x Cc2d1a*^+/+^*, G2* = *Cc2d1a*^+/−^ *x Cc2d1a*^+/−^, and *control* = *Cc2d1a*^+/+^ *x Cc2d1a*^+/+^. Fold-change values for six differentially expressed miRNAs are illustrated by the variation in color for the 5p strand (6) and the 3p premicroRNAs (5) in the sperm (**E**), blood (**F**), and hippocampus (**G**) of the examined groups. In the figure, the columns represent groups, and the rows represent miRNAs (red, black and green correspond to upregulated, unchanged and downregulated, respectively).

Two-week-old mice have not yet completed brain development, and their hippocampal and cerebellar granule cells continue to migrate and differentiate^26–28^ and VPA-treated males will develop the characteristic alterations in behavior. The spermatogonial stem cells that will continuously divide and differentiate throughout the life of the male are present, leaving open the possibility that some changes in transcript levels may be transmitted to the next generation. While concentrations of 600 mg/kg and higher were found to be lethal in both genetic backgrounds (Supplementary Table 3), lower concentrations did not affect body weight or cause any visible phenotype in the mice, such as the previously reported “crooked tail” phenotype^24^. After the injection of 500 mg/kg VPA, a few males became aggressive toward females during breeding, showed reduced fertility and smaller testes, and were not studied further. The 500 mg/kg dose of valproic acid was reproducibly found optimal for both lines of mice. The exhibited homogenous phenotypes, including characteristic behavior changes and miRNA transcript analysis results are presented only for the *Balb/c* line in Figs. 3 and 4 of controls and VPA treated 500 mg/kg.

#### *Cc2d1a*^+/−^ heterozygous mice

Mice carrying a *Cc2d1a* knock-out mutation have been reported to present embryonic brain impairment resemble to ASD characteristics^29^. We purchased mice heterozygous for this mutation (*Cc2d1a*^+/−^ mice) from the Jackson Laboratory and outbred them into the *Balb/c* background for 10 generations before the initiation of the experimental procedures. Homozygotes (−/−) show lethality beginning at 14 dpc and extending to different embryonic developmental stages in the *Balb/c* line, and no living homozygotes are observed. Heterozygotes (+/−) were established and maintained, as verified by PCR genotyping, according to the instructions of Jackson Laboratory (see Methods and Supplementary Fig. 2 for PCR genotyping). *Cc2d1a*^+/−^ males were mated with normal (+/+) or heterozygous (+/−) females, successively generating G1 and G2 generations.

Further analysis of both models was conducted on groups of two-month-old males (10 males mice) from all groups (Supplementary Tables 3 and 4), including *Balb/c* males controls (controls are laboratory mice never been crossed with *Cc2d1a*^+/−^ mutants mice) subjected to behavioral tests including recognition of a novel versus a familiar object, social interactions, tail suspension and marble burying (see Fig. 3 for the experimental timeline). All analyses were performed in a blinded manner. The results of the behavioral tests and the statistical significance reported in Fig. 3 clearly show the development of altered behavior in the mouse models compared to the control groups that could be related to ASD-like characteristics. The behavioral tests were performed as described in the Methods and Supplementary Data sections and Supplementary Tables 3 and 4. The same approach could be extended in the future to the others ASD related gene candidates in mouse models.

### Behavioral observations

#### Novel object test

Object recognition was measured as shown in Fig. 3A,B, according to the difference in the time spent with familiar versus new objects (see Materials and Methods)^15–17,30–32^. In fact, all control groups (untreated or treated with peritoneally injected saline) showed more interest in novel objects than familiar objects. The VPA-treated (500 mg/kg) males used as positive controls lost interest and spent the same amount of time with the two objects but spent less time overall with both objects; i.e., there was little distinction between the two objects. Some effect was detectable at a dose of 400 mg/kg, but only for familiar objects. Thus, the effect of VPA was dependent on the dose. In addition, there was a significant difference between the control and VPA groups in the time spent with familiar and new objects and the number of visits (Fig. 3A). A decrease in the overall activity as well by half was also observable in the open field and Morris water tests in the 500 mg/kg VPA group (not shown). Two types of genotypes heterozygous and wild type (living mice) are produced under crossing protocol of G1 (*Cc2d1a*^+/−^ males were mated with normal (+/+) females) and G2 (*Cc2d1a*^+/−^ males were mated with *Cc2d1a*^+/−^ females). From both group (G1 and G2) all genotypes are separately analyzed and here the males with wild type genotype are slightly different from male controls group (males never crossed with *Cc2d1a*^+/−^ genotype). In the *Cc2d1a*^+/−^ group (Fig. 3D), the effect was slight but still observable. In fact, slightly less interest in the novel object was observed in the test group compared to the controls, especially in the G1 group. However, some minor differences in examination behavior were observed between the model and controls groups.

#### Social interaction test

The three-chamber social interaction test was originally adapted from Crawley’s group and was slightly simplified (see Materials and Methods)^30,33^. Social interaction was measured as shown in Fig. 3C,D, according to the difference in the time spent close to a chamber containing one living mouse compared to an empty chamber. In fact, almost all of the control groups (untreated or treated with peritoneal saline injection) showed no difference in the interaction between the two chambers. The VPA-treated (500 mg/kg) males, used as a positive control, generally showed less interest and spent an even smaller amount of time close to the empty cage (Fig. 3C). Again, the 500 mg/kg VPA results showed marked differences. We took into account all tests involving 500 mg/kg dose-induced phenotypes. A decrease in overall activity in the 500 mg/kg VPA group was also detectable in social interaction tests. In contrast, in the *Cc2d1a*^+/−^ group (Fig. 3D), we observed a marked difference in social interaction in which more time was spent close to the empty cage rather than the cage with a living mouse. The same effect was observed in the G1+/− and +/+ and G2+/− groups. In contrast, no difference was detected in the G2+/+ test group compared to the controls. The *Cc2d1a*^+/−^ group did not behave similarly to the VPA group in the social interaction tests, but variation from the control group (never crossed to *Cc2d1a*^+/−^ genotype) confirmed behavioral changes. The wide variation in ASD phenotypes observed herein in the two ASD-like mouse models compared with the controls is also relevant.

#### Tail suspension test

In this simple test, a mouse was suspended by its tail with tape, and the movements of the animals in the air were recorded (see Materials and Methods)^30,34^. The total duration was six minutes, and struggle-related behaviors were assessed. Periods of agitation and immobility were recorded. At the 500 mg/kg dose of VPA, a significant change that indicated abandonment and depression was observed (Fig. 3E). In these tests, we observed that the effect was significantly different from what was observed in the controls, but the reverse effect was observed in the F1 VPA group compared to the F0 VPA generation. In the G1 and G2 *Cc2d1a*^+/−^ groups (Fig. 3F), slight variation from the control was observed but in an opposite direction from the VPA 500 mg/kg behavior. Non-significant differences in tail suspension were observed in the *Cc2d1a*^+/+^ group (G1 and G2).

#### Marble burying (MB) test

Repetitive action was evaluated in the marble burying test^35^. Twenty glass marbles were placed on the surface of clean bedding, and the number of marbles buried in 30 minutes was scored as described in the Materials and Methods. VPA-treated (500 mg/kg) males (Fig. 3G), used as positive controls, again generally showed markedly less interest in burying marbles. In the 500 mg/kg VPA F1 generation, we observed the same behavior as in the founders, with significantly less activity. In contrast, in the *Cc2d1a*^+/−^ group (Fig. 3H), we observed slightly more burying activity than in the controls group (never crossed to *Cc2d1a*^+/−^genotype).

### miRNA analysis of the mouse models

The six-microRNAs that had been found downregulated in patients were affected to a comparable extent in the two mouse models with altered behavioral traits characteristic of the ASD phenotype Fig. 4 and Supplementary Fig. 3. Confirming the association of the observed molecular effects with the disease and paving the way for further investigations not possible in human subjects for obvious practical, ethical and etiological limitations. The low levels of expression of the six-microRNAs downregulated in human sera were paralleled by decreases not only in the blood but also in the sperm and hippocampus of the VPA-treated and *Cc2d1a* mutant mice group (Fig. 4A–C and Supplementary Fig. 3a–d), with marked differences noted as a subject for future enquiries.

We determined that it was not only the 5p strand sequences but also the 3p (premicroRNA) sequences of the 6 microRNAs that were down-regulated in all cases (Fig. 4A,B and Supplementary Fig. 3a–d), The mature and premicroRNA transcripts of a given microRNA share common functional connections, but the downregulation of both strands strongly suggests an alteration in the initial step(s) generating the transcript.

## Discussion

Given the complexity of the human brain, the deviant mechanisms at the origin of pathologies clinically defined in functional terms are likely to be themselves multiple. It therefore appeared that it would be rewarding to detect a common genetic abnormality in a series of 45 children with autism and their proximal family, including their fathers, mothers, sisters and brothers. Among the 280 microRNAs tested, six (miR-19a-3p, miR-361-5p, miR-3613-3p, miR-150-5p, miR-126-3p, and miR-499a-5p) were significantly downregulated in the sera of children with autism and their relatives but not in a total of 37 control children or genetically unrelated members of ASD-free families. We can also exclude the possibility that in our studies multiplex families would behave as a dominant model suggesting the presence of a genetic predisposition as opposed to sporadic cases. Because, both multiplexes as well as simplex family patients present lower level of six miRNAs to the parents, this is a validation step in unrelated and sporadic cases of ASD. These findings initially appeared significant; with, however, some initial doubt because no such effect on these microRNAs had been reported in previous studies performed on larger cohorts of affected adults and children (reviewed by Hu *et al*.^9,36^). Nevertheless, we are confident regarding the significance of these downregulatory effects as intrinsic components of the ASD pathology because of their systematic occurrence in the 45 patients of the group and further confirmation by identical observations in two recognized animal models of the disease.

A further development of interest was that the observations made in the patients and their closely related family members (siblings and progenitors) define two phenotypes always in a coordinated manner for the six miRNAs: a nearly complete disappearance (90 percent or more) in all the affected patients and a partial decrease (average 40-50 per cent) in their close relatives. Either one or both of the genitors of all the children with ASD represented in the cohort belonged to the second category. These observations therefore define a disease-associated state with an apparently normal phenotype. Although it is not possible to construct physiological models at this stage, the relationship of the effect with the disease is clear, defining a heritable high-risk state leading to the development of actual symptoms in the progeny. It was consistently observed (Fig. 1) that as long as the disease was not clinically evidenced (as in parents of sick children and sibling), intermediate expression levels of the miRNAs were recorded, in contrast with the much lower levels observed in both human patients and affected mice. There is one significant exception with the miR-126-3p in mothers of patients, which is going in the opposite direction, with a +3.985 fold-change. In fact, levels of estradiol are known to be positively associated with miR-126-3p expression^37,38^. The suggestion that such an effect in patient’s mothers could be related to the serum levels of estradiol need further investigation.

Differences in the microRNA expression in parents and unaffected siblings could be explained by age differences of the patients, by exposure to a hypothetical pollutant “eventually originating the disease” could be more efficiently metabolized in adults than in children. However, the results from our mouse models (homogenous age) exclude age related bias.

**Figure 5:**
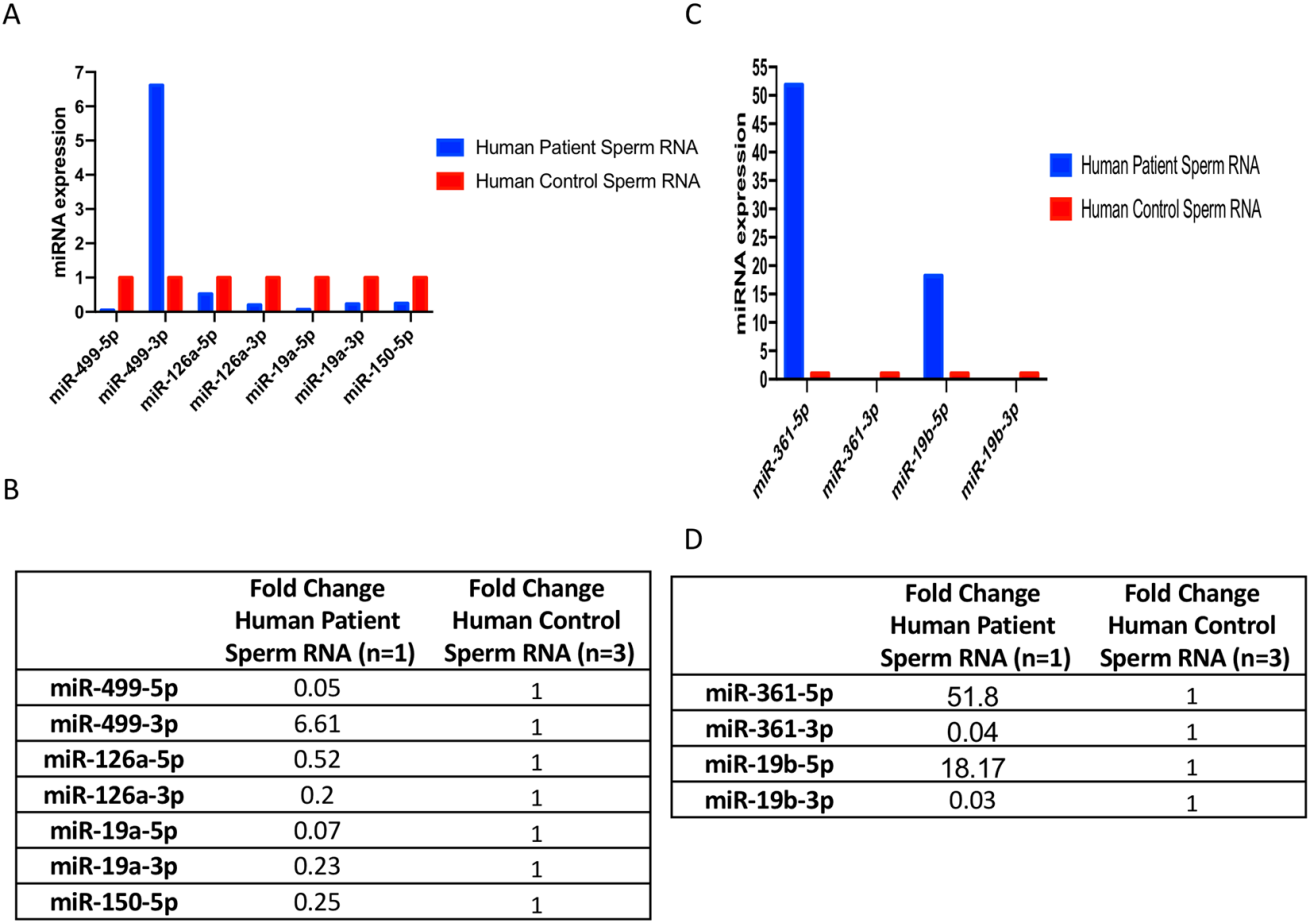
Sperm profiles of the six-miRNAs in the father of three affected children. Sperm profiles of P13F (raw data) is presented for six-miRNAs in a father patient and the three sperm controls. Total sperm RNA was tested by qRT-PCR to determine the levels of the Six-microRNAs. The miRNA transcript profile of human sperm from P13F (the father of three children with distinct behavioral alterations born to different mothers) compared to three sperm sample controls is shown.

The current results suggest a number of questions that can be considered in further research projects. Neither the analysis of gametes nor the assessment of transgenerational maintenance can be performed in human subjects because of obvious practical and ethical limitations, but the murine models showed downregulation of the six miRNAs in their sperm as well as their blood and hippocampus and exhibited transgenerational maintenance of this effect. In humane as an exceptional case, we were able to obtain sperm samples from the father of the three children with behavioral alterations. We observed the downregulation of 5 out of the 6 microRNAs (miR-19a-3p, miR-3613-3p, miR-150-5p, miR-126-3p, and miR-499a-5p microRNAs) Fig. 5 in the father’s sperm compared to 3 independent human sperm controls. The 5p strand and the 3p sequences showed decreased levels in the sperm microRNAs of the father of three children (Fig. 5) as well in mouse test samples (see Fig. 4 in Results section). Such analysis could be easily performed among ASD patients, to confirm test assays and evaluate hereditary issues.

A distinct question to be explored first in animal models is that of a possible epigenetic mode of maintenance and hereditary transmission of the disease-associated states by phenotypically normal progenitors. Remarkably for instance, in the case of the *Cc2d1a*^+/−^ genotype, current preliminary data suggest that the profile of miR expression associated with the disease is maintained in their *Cc2d1a*^+/+^ genotype progeny. *Cc2d1a* is one example of ASD related gene, but the same type of study could be extended to other mice mutants from Jax Mouse laboratory stock. In addition to VPA, several well-known environmental factors exposure either through a mother or after birth are associated with ASD, the corresponding mouse models are required to establish changes in the level of these six microRNAs.

Whether the microRNA profile could be acting per se as a significant transgenerational determinant could potentially be tested by the same approaches as in other epigenetic systems such as microinjection in one-cell embryos^39,40^. The resulting changes can be evaluated at the physiological level starting from the identification of the coding RNAs under the control of the six altered miRNAs. According to the current understanding, the physiological consequence of the low expression of the miRNAs is increased expression of their target mRNAs. Among the predicted targets of the six-miRNAs revealed by computational approaches, there are indeed genes for which abnormal expression ASD patterns have been reported in patients (Supplementary Table 5 and Supplementary Fig. 4). Since both the initial occurrence of the disease and its transgenerational maintenance appear to be correlated with changes in the levels of regulatory microRNAs in somatic tissues and sperm, a general hypothesis that stable expression of their target gene(s) is critical and is determined in the earliest developmental period can be formulated.

In the medical and sociological fields, the current conclusions may lead to positive outcomes for the patients and their families. They provide a critical tool to diagnose the disease at the youngest possible age, even at birth, via a simple, non-invasive, inexpensive examination method, thus allowing children to be provided as early as possible with the proper surroundings to facilitate their development.

## Materials and Methods

### Patient selection criteria

This study was approved by the Ethics Committee of the Erciyes University School of Medicine. A detailed description of the study was provided to all participants and their parents before their enrollment. All parents gave written informed consent before participation (09-20-2011 committee number: 2011/10). The diagnosis was made by a multidisciplinary team (composed of an experienced child psychiatrist, a pediatric neurologist, and a genetic specialist) according to the criteria of the Diagnostic and Statistical Manual, Fourth and Fifth Edition, Text Revision (DSM-IV-TR; American Psychiatric Association, 2000 and DSM-V; American Psychiatric Association, 2013) using the Childhood Autism Rating Scale (CARS) (Schopler, Reichler, DeVellis, & Daly, 1980). All subjects were carefully screened for signs of infection, and subjects with acute illness were excluded. Nine multiplex families (including more than 1 child with an autism diagnosis) and 28 simplex families (including only 1 child with autism) were enrolled in this study. A total of 189 participants were enrolled in the study. We included 45 subjects with ASD (range = 2 to 13 years old; 31 males and 14 females) and 21 age- and sex-matched typical control subjects (range = 3 to 16 years old; 10 males and 11 females). Thirty-three healthy siblings (range = 1 to 20 years old; 17 males and 16 females) were included. All of the participants were of Turkish origin. The ASD group included 27 children with autism spectrum disorder and 18 children with pervasive developmental disorder-not otherwise, specified (PDD-NOS). Twenty-two of the children were diagnosed with intellectual disability, 4 of whom were diagnosed with epilepsy with EEG abnormalities, and 2 of them also exhibited attention deficit hyperactivity disorders (ADHDs) among the autistic cases. Eleven children were diagnosed with mental retardation, 5 of whom were diagnosed with ADHD among the PDD-NOS cases. Patients were excluded if they exhibited genetic disorders, including chromosomal abnormalities, Fragile X Syndrome, tuberous sclerosis, or neurofibromatosis type 1. No clinical or laboratory findings suggesting autism or other diseases were detected in the control group. All patient characteristics are presented in Supplementary Table 1a.

### Blood collection, serum separation and RNA isolation

Blood samples were collected from the donors and healthy controls after obtaining written informed consent from all of the parents. In total, 189 family members (45 autistic patients, 33 unrelated healthy siblings, and 74 parents) and 37 sex- and age-matched healthy control samples were included in the dynamic array to investigate 384 miRNAs. Two-milliliter blood samples were collected from all of the family members and matched controls. Blood samples were collected between 11.00 and 13.00 to eliminate unwanted variation in the examined parameters. All protocols for serum separation were completed within 1 hour of drawing blood. Then, the samples were held for 30 minutes at room temperature. Serum was separated by centrifugation at 3500 rpm for 10 minutes at room temperature. Hemolyzed samples were excluded from the study. The clear supernatant was collected in 200 μl aliquots in new RNase/DNase-free microfuge tubes. RNA was isolated using a High Pure miRNA Isolation Kit (Cat. No: 5080576001; Roche, Mannheim, Germany) according to the manufacturer’s instructions and stored at −80 °C until use.

### cDNA preparation and pre-amplification

Isolated RNA samples were reverse transcribed into cDNA in 5 μl final reaction volumes using a TaqMan microRNA reverse transcription kit (Applied Biosystems, Foster City, CA, USA) as specified in the manufacturer’s protocol. Reverse transcription was performed using a LightCycler 480 Real-Time PCR System (Roche, Mannheim, Germany). cDNA samples were kept at −80 °C until PCR analysis. We performed preamplification after reverse transcription using a TaqMan PreAmp Master Mix 29 system (Applied Biosystems, Foster City, CA, USA) as well as the Megaplex Human Primer Pools Set v3.0 (Applied Biosystems, Foster City, CA, USA). For preamplification, 2 μl of a 1/5-diluted RT product was added to 3 μl of the PreAmp mix. The miRNA TaqMan PreAmp thermal protocol was as follows: 95 °C for 600 sec, 55 °C for 120 sec and 72 °C for 120 sec, followed by 18 cycles of 95 °C for 15 sec and 60 °C for 240 sec, and finally, 99.9 °C for 600 sec. The preamplified cDNA samples were stored at −20 °C for further analysis.

### Quantitative real-time polymerase chain reaction (qRT-PCR)

qRT-PCR was performed by using a high-throughput BioMark Real-Time PCR system (Fluidigm, San Francisco, CA, USA). Preamplified cDNA samples were diluted with low-EDTA (0.1 mM) TE buffer (1:5). Approximately 490 μl of TaqMan Universal PCR Master Mix, No AmpErase UNG (Applied Biosystems, Foster City, CA, USA) and 49 μl of 20X GE Sample Loading Reagent (Fluidigm, San Francisco, CA, USA) were mixed and pipetted into a 96-well plate, and 3.85 and 3.15 μl of 1:10-diluted preamplified cDNA was pipetted into each well and mixed. Then, 5 μl of this mixture was pipetted into the sample inlets of a 96.96 Dynamic Array (Fluidigm, San Francisco, USA), and 4 μl aliquots of 1:1-diluted 209 Assays were pipetted into the assay inlets of a 96.96 Dynamic array (Fluidigm, San Francisco, USA). A BioMark IFC controller HX (Fluidigm, San Francisco, CA, USA) was used to distribute the assay mixture and the sample mixture from the loading inlets into the 96.96 Dynamic array reaction chambers for qRT-PCR by using Fluidigm’s Integrated Fluidic Circuit Technology. The real-time PCR step was performed by using a BioMark System with the following protocol: the thermal mixing protocol was followed by heating at 50 °C for 120 sec, 70 °C for 1,800 sec, and 25 °C for 600 sec. Then, the UNG and hot-start protocol were followed by heating at 50 °C for 120 sec and 95 °C for 600 sec. Finally, PCR was performed with 40 cycles at 95 °C for 15 sec and 60 °C for 60 sec. The BioMark system is quantifies low-abundance miRNAs and can detect a single copy at a Ct value of 26–27.

Routine qPCR for miR detection and validation^41^ (see Supplementary Table 2 for miR sequences) was performed with the miScript PCR control set (catalog number 218380; Qiagen, Germany). The miScript™ miRNA PCR Array Human Serum & Plasma 384HC (Cat No: 331223) was used in this study. The Human Serum & Plasma 384HC miScript miRNA PCR Array profiles the expression of 372 miRNAs (see list of the microRNAs in Supplementary Table 1c) that are detectable in serum and plasma using the miScript PCR system. SNORD61, SNORD68, SNORD72, SNORD95, SNORD96A, RNU6-2, miRTC, miRTC, and PPC were used as controls. The data were normalized using the 2^−∆∆ct^ method.

### Data normalization and analysis

Data were collected using Fluidigm Real-Time PCR Analysis Software, the linear derivative baseline correction method, and the auto global Cq threshold method. System-provided Cq values of 999 and values larger than 26 were considered nonspecific and beyond the detection limits of the system and were therefore removed. Median LOD Cq values were calculated across all arrays to impute missing values. Data normalization was performed using the 2^−ΔΔCT^ method. To detect the differentially expressed miRNAs, linear models for the microarray and RNA-Seq data (limma) were applied. The Benjamini-Hochberg false discovery rate correction was used to adjust *p* values to account for multiple testing. DIANA-mirPath software (Vlachos, 2005) was used to identify signaling pathways based on the differentially expressed miRNAs. Highly accurate and experimentally verified TarBase targets were considered potential targets, and the agglomerative hierarchical clustering algorithm was applied to determine miRNA and pathway clusters based on their interaction levels. The analysis was conducted using the Rcmdr (Fox, J., 2005) and limma^41^ packages of R 3.1.2 software (URL http://www.R-project.org/) and, *p*-values less than 5% were considered to indicate statistical significance.

### Mice

The mice were maintained in a facility under controlled conditions (light from 06:00 to 18:00, 22 °C temperature, 55% humidity). All animals models are followed in the same animal house and time scale, well controlled conditions of food, water, temperature, light and care. Only two persons, always the same are allowed to enter to animal rooms. The animals were cared for and treated according to the Principles of Laboratory Animal Care (European rules). All experiments were approved by the Erciyes University Animal Ethics Committee (04-11-2012) (12/54). All tests were performed between 10:00 and 16:00 in isolated rooms. In all tests, the results for the Balb/c mouse line are presented.

### Genotyping

*Cc2d1a*^+/−^ mice were purchased from the Jackson Laboratory. Heterozygotes were produced by outbreeding in the *Balb/c* background for 10 generations before the initiation of the experimental procedures. Heterozygotes were selected by PCR genotyping according to the instructions for the corresponding Jackson Laboratory^29^ oligonucleotides (CCD1A-M1: 5′-GTG CGA GGC CAG AGG CCA CTT CTG-3′, CCD1A-M2: 5′-GAC CCT GAG AGA GCT CCT GAG AGC-3′, CCD1A-M3: 5′-TT CCC ACC TCT TCT GGC CCA GAG G-3′). The PCR products subjected agarose gel electrophoresis are shown in Supplementary Fig. 2.

### RNA extraction

Total RNA was prepared from mouse tissues (blood, hippocampus, sperm) according to published procedures^42^. Sperm cells were isolated from the epididymis, and floating sperm were recovered by successive washing (PBS) and centrifugation at 3000 rpm. Briefly, RNAs were extracted from all tissues (hippocampus, blood and sperm cells) via a standard protocol. Qiazol Lysis Buffer (Cat. No: 79306; Qiagen, Texas, USA) was used in accordance with the manufacturer’s instructions. The Qiazol-extracted aqueous phase was ethanol precipitated, followed by washing twice with 70% ethanol. To remove the remaining DNA, the PCR samples were treated with DNase according to the manufacturer’s instructions.

Finally, the quantity (absorbance at 260 nm) and quality (ratio of absorbance at 260 nm and 280 nm) of the RNA were evaluated with a BioSpec-Nano spectrophotometer. RNA was stored at −80 °C until use.

### VPA postnatally exposed males

The sodium salt of VPA (Sigma, St. Louis, MO) was prepared in 0.9% saline at concentrations of 300, 400, 500, 600 and 700 mg/ml, pH 7.4 (Supplementary Table 3). The control group received the same volume (0.1 ml) of 0.9% saline buffer. At 14 days post birth, all offspring (8–12 pups from each litter) were injected (intraperitoneally) with VPA at 300 to 700 mg/ml or with saline buffer as a control. Doses of 600 and 700 mg/kg resulted in dramatic lethality, and the animals were therefore not included in the test group. The F1 to F2 generations were obtained following treatment with the 300 to 500 mg/kg VPA doses. The F1 generation derived from VPA-injected F0 fathers is referred to as VPA-F1, while that from saline-injected fathers is identified as the control. Adult VPA-F1 males were bred with normal females.

The control groups were bred under the same housing conditions across all generations. Only male mice were utilized for all experiments.

### Tests of autism-like behaviors in mouse models (VPA-treated group and *Cc2d1a* heterozygotes)

#### Novel object recognition test

The time spent with familiar versus novel objects highlighted a common characteristic of the test group versus the controls. Specifically, it indicated differences in the interest in and recognition of objects. NOR (novel object recognition) is a well-established test in a variety of animal models with multiple protocols (Fig. 3). In general, it involves two trials of cognition evaluation based on the spontaneous exploratory conduct of a mouse to measure recognition memory. In the first trial, we used (first-day acquisition) animals that were exposed to two similar objects (small orange boxes) in a chamber for 5 minutes. During the second trial (second-day retention) mice were again exposed to two dissimilar objects for 5 minutes, including one familiar object from the first trial and one new object (blue box). Object recognition was measured as shown in Fig. 3(A,B), according to the difference in the time spent with the familiar object versus the new object.

#### Social interaction test

Mice grouped in cages exhibit additional behaviors that are referred to as social interactions. To determine whether these interactions varied between the groups of mice, they were filmed for 5 minutes. There are many different ways to follow social interaction. Here, we present the results in a simple way based on conditions in a cage separated into three sections. The mice being tested were placed in the center of the cage and were filmed to calculate the time that they spent close to the empty compartment or the compartment occupied by another mouse. The three chambers were separated by square doors in the two inner walls connecting to the center area. Briefly, the test mouse was introduced in the center to initiate habituation for 5 minutes while blocking access to the side compartments. The first test was conducted by placing an unfamiliar mouse inside an empty wire cage in one of the side chambers to measure the social interaction of the subject mouse without direct social contact. On the other side, there was an empty wire cage. Each test was performed for 10 minutes Fig. 3(C,D), and the floor surfaces were wiped with 70% ethanol between trials. The accumulated time spent in each compartment and the sociability or social preference indices were measured to quantify the social behavior of the mice.

#### Tail suspension test

Adult mice (2 months old) (*Balb/c* strain) weighing 25–27 g on the test day. The apparatus used for the tail test consisted of two filter covers each and enabled three mice to be tested simultaneously. Each mouse was suspended by the tail from a hook connected to the strain gauge, to which they were attached with adhesive tape with a length of 18 cm. The duration of each trial was 360 seconds. The tail suspension test was recorded using a SONY HDR-CX240E video recorder. After the recording, the period of the immobility of the mice (in which they remained inactive) was calculated manually. An immobility posture indicates the abandonment of struggling and, thus, depression. Periods of agitation and immobility are reported in Fig. 3(E,F).

#### Glass marble burying test

According to the described protocols, newly filled bedding cages were prepared with 20 marbles spaced on the bedding surface. Mice were individually caged. After 30 minutes, each mouse was returned to its home cage, and the buried marbles were counted (Fig. 3G,H). Marbles that were not buried were not included in the analysis.

### F1 VPA generation

The next generation of mice was obtained from crosses between VPA-treated males and normal partners. At two months, their behavior was tested and compared to that of the founder males. Variation was observed between the F1 mice and the father, but they were still different from the control group.

### Statistical analysis

All data are presented as the mean and standard deviation (SD). For the statistical analysis of the behavioral test results, an unpaired t-test and one-way ANOVA were performed (in the case of a significant comparison result (p < 0.05), Tukey’s test was used as a post hoc multiple comparison test). The p-value significance level was set at <0.05.

### Ethics statement

This study was approved by the Hospital Ethics Committee and authorizations from all the patients and the participating relatives were obtained by signing an informed consent form. All parents gave written informed consent before participation (09-20-2011 committee number: 2011/10). All research was performed in accordance with the relevant guidelines and regulations (Erciyes University animal ethics committee 04-11-2012 12/54).

## Supplementary information

Dataset 1.

Dataset 2.

## Data Availability

The datasets generated and analyzed during the current study are available from the corresponding author on reasonable request.
