# Supplementary material for: A heritable profile of six miRNAs in autistic patients and mouse models
Source: Sci Rep. 2020 Jun 9;10:9011. doi: 10.1038/s41598-020-65847-8 (PMC7280218; doi:10.1038/s41598-020-65847-8)
Supplement: Supplementary file 2 — Dataset 2. [file 41598_2020_65847_MOESM2_ESM.pdf]

Supplementary Figures 1, 2, 3 and 4
Supplementary Tables 1a,b,c, 2, 3, 4 and 5

A heritable profile of miRNAs in autistic patients and mouse models.

Yusuf Özkul[*1, 2], Serpil Taheri[2, 3], Kezban Korkmaz Bayramov[1, 2], Elif Funda Şener[2, 3], Ecmel Mehmetbeyoğlu[2], Didem Behice Öztop[4], Fatma Aybuğa[2], Esra Tufan[2], Ruslan Bayramov[1], Nazan Dolu[5], Gökmen Zararsız[2], Leila Kianmehr[8], Feyzullah Beyaz[6], Züleyha Doğanyiğit[7], François Cuzin[8], and Minoo Rassoulzadegan[*2, 8]

Supplementary Table 1a EVRENA Clinics

Patients	Gender	Age	Diagnosis	Clinical Findings	ADSI	Family History	Consanguinity
14-101	Female	5	Autism	ID, Epilepsy		None	Positive
14-102	Female	3	Autism	ID, Epilepsy		None	Positive
15-101	Male	9	Autism	ID		Epilepsy, Deafness	Positive
15-102	Male	9	Autism	ID		Epilepsy, Deafness	Positive
16-101	Female	9	Autism	ID		Autism	Positive
16-102	Male	9	Autism	ID		Autism	Positive
16-103	Male	5	Autism	ID		Autism	Positive
17-101	Female	8	Autism	ID		None	Positive
17-102	Female	12	Autism	ID		None	Positive
19-101	Male	5	Autism	ID		MR	Positive
20-101	Male	2	Autism	ID		None	Positive
21-101	Male	12	Autism	ID		Schizophrenia	Negative
22-101	Female	11	Autism	ID		None	Negative
24-101	Male	4	Autism	ID		None	Negative
28-101	Female	13	Autism	ID, Epilepsy		None	Positive
29-101	Male	6	Autism	ID, Epilepsy		None	Positive
31-101	Female	6	Autism	ADHD		Autism	Positive
32-101	Male	3	Autism	ID, Epilepsy	Suspicious	Deafness	Negative
37-101	Male	10	Autism			None	Positive
41-101	Male	5	Autism	ID, ADHD		None	Negative
H10-103	Male	3	Autism	ID	Abnormal	Acromegaly, Deafness	Negative
H10-102	Male	3	Autism	ID	Abnormal	Acromegaly, Deafness	Negative
H13-101	Female	3	Autism	ID		Deafness	Negative
H33-102	Female	3	Autism	ID		Schizophrenia, Asperger	Negative
H34-101	Male	9	Autism			Schizophrenia	Negative
H36-101	Male	9	Autism	ID	Abnormal	None	Positive
H37-101	Male	4	Autism	ID	Abnormal	None	Positive
18-101	Male	6	Atypic Autism		Abnormal	Epilepsy, MS	Negative
25-101	Male	7	Atypic Autism	ADHD	Suspicious	MR	Negative
26-101	Female	3	Atypic Autism			None	Negative
27-101	Male	7	Atypic Autism	ID, ADHD	Suspicious	None	Negative
30-101	Male	7	Atypic Autism	ID	Abnormal	None	Positive
34-101	Male	5	Atypic Autism	ID		None	Negative
35-101	Male	9	Atypic Autism	ID, ADHD		None	Positive
36-101	Female	7	Atypic Autism			None	Negative
38-101	Female	9	Atypic Autism			None	Negative
40-101	Male	5	Atypic Autism	ID, ADHD		None	Negative
42-101	Male	5	Atypic Autism	ID, ADHD		None	Positive
H10-101	Male	6	Atypic Autism	ID, ADHD		Acromegaly, Deafness	Negative
H17-101	Male	6	Atypic Autism	ID, ADHD	Suspicious	None	Positive
H18-101	Male	6	Atypic Autism			None	Negative
H33-101	Male	3	Atypic Autism	ID	Abnormal	Schizophrenia, Asperger	Negative
H34-102	Female	9	Atypic Autism			Schizophrenia	Negative
H38-101	Male	3	Atypic Autism	ID	Abnormal	Autism	Negative
H39-101	Male	3	Atypic Autism	ID	Abnormal	Schizophrenia	Negative
				ID: intellectual disability	(Ankara Developmental Screening Inventory (ADSI)		

Supplementary Table 1c (Dataset A2) Six miRNAs in autism

		Gender	Age	miR-3613-3p	miR-150-5p	miR-126	miR-361	miR-499	miR-19a-3p		miR-19b-3p
Patient Group	14-101	1	5	0,06406016	2,20436693	0,10378127	0,10378127	0,10378127	0,17188982		0,05181788
	14-102	1	3	0,05183509	1,70563975	0,0253037	0,0253037	0,0253037	0,18251523		0,08271114
	15-101	2	9	0,02703542	0,90787173	0,00557164	0,00557164	0,00557164	0,08868302		0,04143338
	15-102	2	9	0,02223796	0,60688611	0,00497597	0,00497597	0,00497597	0,16068104		0,07086687
	16-101	1	9	0,02592508	2,58633445	0,01627575	0,01627575	0,01627575	0,19574366		0,10221683
	16-102	2	9	0,07373669	2,11725889	0,01006427	0,01006427	0,01006427	0,35160326		0,15456666
	16-103	2	5	0,02225603	0,62742476	0,00352716	0,00352716	0,00352716	0,10052391		0,04095602
	17-101	1	8	0,1571635	4,13455503	0,00845692	0,00845692	0,00845692	0,19372711		0,06344724
	17-102	1	12	0,01436594	0,63921094	0,01768081	0,01768081	0,01768081	0,14757933		0,05847534
	18-101	2	6	0,05406631	1,37404788	0,04108989	0,04108989	0,04108989	0,171538		0,06972576
	19-101	2	5	0,02024567	0,41708861	0,00673725	0,00673725	0,00673725	0,2200476		0,07084169
	20-101	2	2	0,18920363	4,60697688	0,02579342	0,02579342	0,02579342	0,0743006		0,04332998
	21-101	2	12	0,00628218	0,11544325	0,02042128	0,02042128	0,02042128	0,12718349		0,08001071
	22-101	1	11	0,06841813	2,13877521	0,01287916	0,01287916	0,01287916	0,15046541		0,04463947
	24-101	2	4	0,0496596	1,9869573	0,01100886	0,01100886	0,01100886	0,14518914		0,06582181
	25-101	2	7	0,06335224	1,65826286	0,00878297	0,00878297	0,00878297	0,12359312		0,07274616
	26-101	1	3	0,03080977	1,22932197	0,00785318	0,00785318	0,00785318	0,23368638		0,11325856
	27-101	2	7	0,0684967	2,38892718	0,00531768	0,00531768	0,00531768	0,0920413		0,040898
	28-101	1	13	0,04506578	2,16742512	0,01676802	0,01676802	0,01676802	0,25929362		0,15995814
	29-101	2	6	0,08123046	2,63195315	0,0070298	0,0070298	0,0070298	0,17358076		0,09150242
	30-101	2	7	0,08769838	1,97088008	0,01161455	0,01161455	0,01161455	0,13673016		0,09372314
	31-101	1	6	0,03293949	0,93387114	0,00765101	0,00765101	0,00765101	0,19497356		0,09576237
	32-101	2	3	0,13598872	4,34917405	0,00567969	0,00567969	0,00567969	0,15370959		0,06989581
	34-101	2	5	0,10938197	2,40437961	0,02171658	0,02171658	0,02171658	0,16444628		0,05804484
	35-101	2	9	0,07817531	1,88637604	0,02432375	0,02432375	0,02432375	0,08505344		0,04599123
	36-101	1	7	0,05853323	2,0820171	0,01130222	0,01130222	0,01130222	0,13718678		0,07247137
	37-101	2	10	0,10642895	2,85530039	0,00691214	0,00691214	0,00691214	0,11281774		0,04150773
	38-101	1	9	0,11262978	2,69477488	0,00640568	0,00640568	0,00640568	0,22097763		0,11660454
	40-101	2	5	0,01703726	0,6055294	0,02590442	0,02590442	0,02590442	0,15676286		0,05530055
	41-101	2	5	0,04714414	0,66309917	0,00830172	0,00830172	0,00830172	0,13040654		0,06028809
	42-101	2	5	0,10793472	4,02135633	0,00472728	0,00472728	0,00472728	0,2048886		0,09954918
	H10-101	2	6	0,07183464	1,93421175	0,00350714	0,00350714	0,00350714	0,3376697		0,15964515
	H10-103	2	3	0,14631391	1	0,06164948	0,06164948	0,06164948	0,07841382		0,03357094
	H10-102	2	3	0,22676724	4,37973319	0,00854276	0,00854276	0,00854276	0,08050185		0,04553324
	H13-101	1	3	0,07017103	1,96044275	0,00618775	0,00618775	0,00618775	0,15252761		0,06968147
	H17-101	2	6	0,00560289	0,21013914	0,01354069	0,01354069	0,01354069	0,1451336		0,05559482
	H18-101	2	6	0,03385364	0,3771845	0,04562889	0,04562889	0,04562889	0,15804148		0,07391515
	H33-101	2	3	0,11191796	3,02611661	0,00561406	0,00561406	0,00561406	0,16367799		0,07595937
	H33-102	1	3	0,03788721	0,25894468	0,05912833	0,05912833	0,05912833	0,11083659		0,02691006
	H34-101	2	9	0,09029278	3,15090835	0,00985427	0,00985427	0,00985427	0,26107852		0,11862139
	H34-102	1	9	0,06644555	3,42081726	0,01052233	0,01052233	0,01052233	0,20114483		0,09471678
	H36-101	2	9	0,09111909	1,70551387	0,01461379	0,01461379	0,01461379	0,41211908		0,18017071
	H37-101	2	4	0,04075513	1,12602846	0,01036059	0,01036059	0,01036059	0,54408269		0,19840897
	H38-101	2	3	0,04891729	0,93630387	0,00862369	0,00862369	0,00862369	0,20263398		0,09188679
	H39-101	2	3	0,02156883	0,4449249	0,01108614	0,01108614	0,01108614	0,27384518		0,1111823
Brother Group	17-103	1	16	0,09916185	2,7276856	0,00513333	0,00513333	0,00513333	0,30613472		0,13026877
	17-104	1	14	0,04445609	1,163651	0,00508093	0,00508093	0,00508093	0,30043483		0,10880005
	18-102	2	11	0,02764692	0,36830906	0,00832085	0,00832085	0,00832085	0,10016462		0,04755698
	18-103	2	4	0,11631301	2,69241231	0,02634922	0,02634922	0,02634922	0,15573991		0,05639171
	19-102	1	20	0,02019464	0,93003545	0,01838462	0,01838462	0,01838462	0,04056981		0,01359994
	19-103	2	15	0,13183873	4,33194363	0,01443048	0,01443048	0,01443048	0,17971104		0,0969249
	22-102	1	9	0,04013194	1,30746917	0,01202031	0,01202031	0,01202031	0,08656953		0,08672916
	25-102	2	17	0,02102661	0,15073047	0,02157062	0,02157062	0,02157062	0,14714664		0,06753718
	25-103	1	15	0,07533637	2,35886517	0,00384043	0,00384043	0,00384043	0,1034804		0,04312016
	25-104	1	10	0,03805495	1,27708059	0,01025186	0,01025186	0,01025186	0,26966952		0,11648529
	26-102	1	8	0,08294906	2,28449671	0,01062469	0,01062469	0,01062469	0,22501323		0,12904416
	27-102		14	0,0832809	2,16590709	0,0130466	0,0130466	0,0130466	0,16485114		0,08551939
	28-102	1	17	0,02907263	0,96743125	0,00242797	0,00242797	0,00242797	0,2145673		0,08730845
	28-103	2	15	0,06418854	1,14088431	0,01101625	0,01101625	0,01101625	0,15592068		0,06476882
	28-104	2	4	0,18643994	5,60170638	0,01637736	0,01637736	0,01637736	0,23666842		0,13642286
	29-102	2	10	0,1879068	6,01826548	0,01045124	0,01045124	0,01045124	0,13746629		0,0488351
	30-102	2	6	0,07197544	1,60800887	0,00348697	0,00348697	0,00348697	0,13497917		0,0544708
	31-102	1	13	0,09359738	2,23744832	0,01207203	0,01207203	0,01207203	0,12760744		0,05500023
	31-103	2	7	0,07846727	1,91775331	0,00879441	0,00879441	0,00879441	0,15126928		0,07685648
	31-104	2	7	0,01696669	0,4630876	0,00860106	0,00860106	0,00860106	0,13269224		0,04739728
	34-102	1	19	0,04680744	1,17339033	0,01719002	0,01719002	0,01719002	0,11323451		0,05578425
	34-103	2	14	0,02361663	1,09578849	0,01064172	0,01064172	0,01064172	0,24903035		0,12179313
	35-102	1	7	0,02060764	0,67295349	0,01887485	0,01887485	0,01887485	0,1326653		0,08585888
	36-102	2	3	0,02154046	0,55681642	0,02904697	0,02904697	0,02904697	0,25331644		0,1257037
	37-102	1	9	0,01740056	0,44235943	0,01815524	0,01815524	0,01815524	0,12677606		0,04881251
	37-103	1	7	0,02169391	0,78222008	0,00232422	0,00232422	0,00232422	0,19930196		0,09895752
	37-104	1	5	0,08742603	2,26026833	0,03609609	0,03609609	0,03609609	0,44585957		0,14762908
	37-105	2	1	0,05880055	1,46305647	0,01260586	0,01260586	0,01260586	0,21438158		0,08564824
	42-102	1	10	0,03818084	0,87542459	0,00901873	0,00901873	0,00901873	0,12407704		0,08009269
	H13-102	2	14	0,04840538	1,19641419	0,00774369	0,00774369	0,00774369	0,15995802		0,09764762
	H13-103	2	18	0,06078119	1,11471184	0,00516054	0,00516054	0,00516054	0,19729635		0,06675219
	H18-102	2	9	0,03609	2,0778987	0,01382294	0,01382294	0,01382294	0,18283827		0,08162268
	H36-102	2	17	0,04877062	1,28760083	0,0047208	0,0047208	0,0047208	0,2478237		0,10510048
Father Group	14-201	2	32	0,01067789	0,33709655	0,04027794	0,04027794	0,04027794	0,22086644		0,11256476
	15-201	2	33	0,03567133	0,47629837	0,00764364	0,00764364	0,00764364	0,19737893		0,11243721
	16-201	2	38	0,12100667	1,41540761	0,00977993	0,00977993	0,00977993	0,14639678		0,05880947
	17-201	2	31	0,0812018	1,29889253	0,01304039	0,01304039	0,01304039	0,07073929		0,03394013
	18-201	2	41	0,04007834	1,13949484	0,01078971	0,01078971	0,01078971	0,1474109		0,06614839
	19-201	2	46	0,19903267	3,11999405	0,01996804	0,01996804	0,01996804	0,2074587		0,08951037
	20-201	2	31	0,08968288	1,8796634	0,04602622	0,04602622	0,04602622	0,34984136		0,1438464
	21-201	2	38	0,04611353	1,42861682	0,0164291	0,0164291	0,0164291	0,02471356		0,01165494
	22-201	2	35	0,05475405	1,13996991	0,02055925	0,02055925	0,02055925	0,11571268		0,05838982
	24-201	2	30	0,10980819	1,72190068	0,02270648	0,02270648	0,02270648	0,15358671		0,06504153
	25-201	2	40	0,06834957	1,60224496	0,01041907	0,01041907	0,01041907	0,0908087		0,04491859
	26-201	2	31	0,33971305	7,01483354	0,01069581	0,01069581	0,01069581	0,18156041		0,08830623
	27-201	2	41	0,01798982	0,64169377	0,00795094	0,00795094	0,00795094	0,15250133		0,06141055
	28-201	2	38	0,01612258	0,76926505	0,02504832	0,02504832	0,02504832	0,1243257		0,04724399
	29-201	2	27	0,00917248	0,59172729	0,01496955	0,01496955	0,01496955	0,15148475		0,04675066
	30-201	2	30	0,06589311	1,24102039	0,0153193	0,0153193	0,0153193	0,11828422		0,03347546
	31-201	2	38	0,04670409	1,37735993	0,01093642	0,01093642	0,01093642	0,12559727		0,04537709
	32-201	2	30	0,27048865	6,24771527	0,0756065	0,0756065	0,0756065	0,20313373		0,24557731
	34-201	2	47	0,0747095	2,22798113	0,03564228	0,03564228	0,03564228	0,40878619		0,09158188
	35-201	2	40	0,06668177	3,19153438	0,05003755	0,05003755	0,05003755	0,35543271		0,11006779
	36-201	2	31	0,14844642	3,28940273	0,02017875	0,02017875	0,02017875	0,25751181		0,15863141
	37-201	2	31	0,17280713	3,76560801	0,02315332	0,02315332	0,02315332	0,4683798		0,21432582
	38-201	2	38	0,07481971	2,87423289	0,0781386	0,0781386	0,0781386	0,63677558		0,34618307
	40-201	2	48	0,22997558	6,46160145	0,04326039	0,04326039	0,04326039	0,55373716		0,3051089
	41-201	2	40	0,22597596	5,47921756	0,04378263	0,04378263	0,04378263	0,32081903		0,12670861
	42-201	2	32	0,10632762	4,31093109	0,03144772	0,03144772	0,03144772	0,49903615		0,23565337
	H10-201	2	35	0,23237079	10,710607	0,06989793	0,06989793	0,06989793	0,37030786		0,17246241
	H10-202	2	33	0,10725056	2,92161994	0,02506931	0,02506931	0,02506931	0,34722488		0,16170653
	H13-201	2	42	0,2906643	4,5055297	0,10556029	0,10556029	0,10556029	0,58000418		0,33856769
	H17-201	2	33	0,11385211	0,88910363	0,03365885	0,03365885	0,03365885	0,43015957		0,11696199
	H18-201	2	38	0,23998855	8,63703819	0,04390012	0,04390012	0,04390012	0,36940684		0,13238418
	H33-201	2	32	0,55896825	12,2109885	0,0418582	0,0418582	0,0418582	2,26849429		1,19336175
	H34-201	2	34	0,18849484	4,96700787	0,03474941	0,03474941	0,03474941	0,55725654		0,33623229
	H36-201	2	35	0,46326226	17,1345856	0,06359949	0,06359949	0,06359949	1,03455155		0,53760549
	H37-201	2	32	0,43085994	12,0489417	0,02159539	0,02159539	0,02159539	0,81130389		0,34553128
	H38-201	2	33	0,08899668	1,95026399	0,04937852	0,04937852	0,04937852	0,60703088		0,42245378
	H39-201	2	30	0,07904993	1,95369266	0,17990679	0,17990679	0,17990679	2,17341335		0,96443273
Mother Group	14-301	1	31	0,05453253	1,56099341	0,02573502	0,02573502	0,02573502	0,50299486		0,30788067
	15-301	1	33	0,13478955	3,44260208	0,04663671	0,04663671	0,04663671	0,2683306		0,16937239
	16-301	1	39	0,04920611	0,52611482	0,26660007	0,26660007	0,26660007	0,19919234		0,16433249
	17-301	1	35	0,07112851	2,32982037	0,13725371	0,13725371	0,13725371	0,89673856		0,35847674
	18-301	1	31	0,08120223	0,78890575	0,03575804	0,03575804	0,03575804	0,10018409		0,05513403
	19-301	1	40	0,17903074	5,94702792	0,0318384	0,0318384	0,0318384	1,16428854		0,43571723
	20-301	1	22	0,0731085	2,54365635	0,0600218	0,0600218	0,0600218	0,05563817		0,08959683
	21-301	1	37	0,13704169	4,59406589	0,03070351	0,03070351	0,03070351	0,34622459		0,23808615
	22-301	1	31	0,11001154	2,80439629	0,04491368	0,04491368	0,04491368	0,85632162		0,45486786
	24-301	1	24	0,04798273	1,0058038	0,04752873	0,04752873	0,04752873	0,27420777		0,09997163
	25-301	1	38	0,12396779	3,18464761	0,06082218	0,06082218	0,06082218	0,31977398		0,35722239
	26-301	1	29	0,09352563	0,54882781	0,12997531	0,12997531	0,12997531	0,30005599		0,21478859
	27-301	1	46	0,06636768	1,17581795	0,02951478	0,02951478	0,02951478	0,12373558		0,14151056
	28-301	1	33	0,10059397	1,42419369	0,06645587	0,06645587	0,06645587	0,1453074		0,10119171
	29-301	1	30	0,04918758	2,06105554	0,04784711	0,04784711	0,04784711	0,50262289		0,14778763
	30-301	1	22	0,14589901	3,07659321	0,06177194	0,06177194	0,06177194	0,39966484		0,16727356
	31-301	1	38	0,34109821	3,26460651	0,0362204	0,0362204	0,0362204	0,51922754		0,20532932
	32-301	1	24	0,20978822	4,49854847	0,04305107	0,04305107	0,04305107	0,19278676		0,10271851
	34-301	1	40	0,31956271	17,9214425	0,10231249	0,10231249	0,10231249	0,58709876		0,39490193
	35-301	1	35	0,18876128	2,66610668	0,03289652	0,03289652	0,03289652	0,35306705		0,25185204
	36-301	1	28	0,15551823	4,96116329	0,05588496	0,05588496	0,05588496	0,63695132		0,21742164
	37-301	1	35	0,28775265	8,40565219	0,05066637	0,05066637	0,05066637	0,32095122		0,09824508
	38-301	1	34	0,31142493	8,94051895	0,05256287	0,05256287	0,05256287	0,71101536		0,37708356
	40-301	1	37	0,36354844	6,60554254	0,05788654	0,05788654	0,05788654	1,06837248		0,67594221
	41-301	1	33	0,57629888	12,3095913	0,05014685	0,05014685	0,05014685	2,10133748		1,09379971
	42-301	1	30	0,32123615	4,42954828	0,02196751	0,02196751	0,02196751	0,37038023		0,1911205
	H10-301	1	33	0,05648824	2,48973179	0,04887845	0,04887845	0,04887845	0,44058715		0,25451748
	H10-302	1	28	0,2671478	5,21261587	0,10456392	0,10456392	0,10456392	0,72766838		0,27885841
	H13-301	1	34	0,10352318	4,24210242	0,03217687	0,03217687	0,03217687	0,28656375		0,05000457
	H17-301	1	32	0,17565352	6,98918838	0,08830549	0,08830549	0,08830549	0,67511925		0,35688421
	H18-301	1	36	0,19331334	6,56866604	0,07777139	0,07777139	0,07777139	1,15942908		0,28717858
	H33-301	1	31	0,15499877	4,7149512	0,03593744	0,03593744	0,03593744	0,30868239		0,21676457
	H34-301	1	38	0,19719582	4,81596707	0,02417506	0,02417506	0,02417506	0,3317542		0,2147141
	H36-301	1	33	0,24729569	8,10354099	0,03570557	0,03570557	0,03570557	0,62706313		0,36749767
	H37-301	1	27	0,27369041	5,24164058	0,01268405	0,01268405	0,01268405	1,31128646		0,66123832
	H38-301	1	32	0,37883292	14,2194528	0,03829423	0,03829423	0,03829423	1,60536111		0,68458992
	H39-301	1	27	0,3956226	5,61359739	0,05498914	0,05498914	0,05498914	0,43274984		0,24150803
Control Group	K38	2	14	0,75847676	22,9309932	0,01480365	0,01480365	0,01480365	1,09262748		0,58195793
	K39	2	8	0,20934992	5,21741515	0,03513792	0,03513792	0,03513792	1,10801979		0,49838973
	K40	1	6	0,07570774	1,22515409	0,0255138	0,0255138	0,0255138	0,61031468		0,33146499
	K41	2	16	0,37183604	14,1550918	0,04699759	0,04699759	0,04699759	0,92750234		0,35995509
	K42	2	11	0,17216281	6,89811012	0,06569178	0,06569178	0,06569178	1,24012512		0,65566138
	K43	2	10	0,14069603	3,96220636	0,05846127	0,05846127	0,05846127	0,88990389		0,52434519
	K44	2	14	0,2048847	9,44324086	0,04101792	0,04101792	0,04101792	0,47147147		0,27281323
	K45	1	9	0,76909021	18,3427363	0,07565522	0,07565522	0,07565522	1,12309834		0,35106541
	K46	1	9	0,28724141	9,57380644	0,02550749	0,02550749	0,02550749	1,65523746		0,80147884
	K47	1	16	0,20828261	7,18841416	0,13705039	0,13705039	0,13705039	0,17714548		0,02294931
	K48	2	8	0,69855005	22,0185289	0,08196562	0,08196562	0,08196562	0,31667779		0,16131342
	K50	1	14	0,10543854	5,8469258	0,08608266	0,08608266	0,08608266	0,19804814		0,10574641
	K51	1	8	0,46363794	8,53705612	0,09835736	0,09835736	0,09835736	0,17863167		0,0559242
	K52	1	5	0,21956562	7,89489401	0,11672093	0,11672093	0,11672093	0,16346183		0,03574414
	K54	1	11	0,58192334	18,5897784	0,07334526	0,07334526	0,07334526	1,29875234		0,64095401
	K55	1	8	0,16951102	2,54507148	0,06312082	0,06312082	0,06312082	0,1574119		0,08155453
	K56	1	5	0,96770222	22,6605225	0,10463207	0,10463207	0,10463207	1,6348792		0,80786807
	K58	2	12	0,68491421	20,234732	0,02150825	0,02150825	0,02150825	0,41129518		0,25950337
	K59	1	9	0,38259097	6,04228038	0,1701233	0,1701233	0,1701233	0,60813106		0,4335325
	K60	2	5	0,17890973	3,44690156	0,06370336	0,06370336	0,06370336	1,54049738		1,10837733
	K61	2	3	0,18440944	5,22873825	0,05302272	0,05302272	0,05302272	1,35938738		0,8569963
	K89	1	32	0,14447688	7,30876818	0,04155005	0,04155005	0,04155005	1,2008678		0,73498189
	K91	1	25	0,28356419	6,3484826	0,16908499	0,16908499	0,16908499	0,40908998		0,33980968
	K94	1	23	0,22107741	3,65891067	0,0587717	0,0587717	0,0587717	0,95266655		0,56584525
	K96	1	33	0,37041472	12,1066326	0,08827788	0,08827788	0,08827788	0,90453106		0,42530009
	K97	1	23	0,19156013	4,62483836	0,01985804	0,01985804	0,01985804	1,31604962		0,59124566
	K98	2	34	0,44979454	9,92240248	0,05550729	0,05550729	0,05550729	2,04072363		1,04796443
	K100	2	23	0,5336144	12,8842986	0,06035019	0,06035019	0,06035019	1,15837345		0,59091557
	K101	1	33	0,25101251	11,7488946	0,08461498	0,08461498	0,08461498	1,44064799		0,7672073
	K103	2	30	0,45056602	5,44214709	0,06454239	0,06454239	0,06454239	0,89417819		0,54497352
	K104	2	32	0,42383826	9,75374932	0,03517292	0,03517292	0,03517292	1,07612564		0,44589696
	K105	2	49	0,55379535	13,7243318	0,03793872	0,03793872	0,03793872	1,63322675		0,78906464
	K106	2	30	0,49608441	9,21219519	0,04010467	0,04010467	0,04010467	0,45922354		0,16576693
	K107	2	50	0,31033116	6,60410106	0,03333764	0,03333764	0,03333764	1,12004783		0,58221645
	K109	2	31	1	1	1	1	1	1		1
	K110	2	33	0,69383532	16,7396901	0,04781826	0,04781826	0,04781826	0,4778918		0,32649652
	K90	1	24	0,13865753	1,41147122	0,05539657	0,05539657	0,05539657	0,36323952		0,24030868
SPERM-RNA	SPERM-RNA	2	42	0,12317476	8,20131866	0,09834269	0,09834269	0,09834269	0,73893351		0,46066153
ref hasta	K109	2	31	1	1	1	1	1	1		1

Supplementary Figure 1 The serum miRNA expression profiles.
($p<0.05$), (log fold change, Unpaired t-test).

A

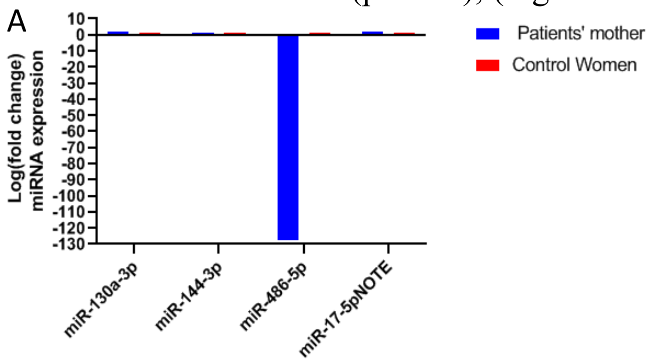

Fig.1a Patients' mothers compared to healthy female control.
4 miRNAs (downregulated) were found as shown in the graph (A) and table (B).

B

	miR-130a-3p	miR-144-3p	miR-486-5p	miR-17-5p
Log(fold change) Patients' mother (n=37)	-0.08926	-0.78616	-127.57338	-0.3695
Log(fold change) Control Women (n=8)	1	1	1	1

C

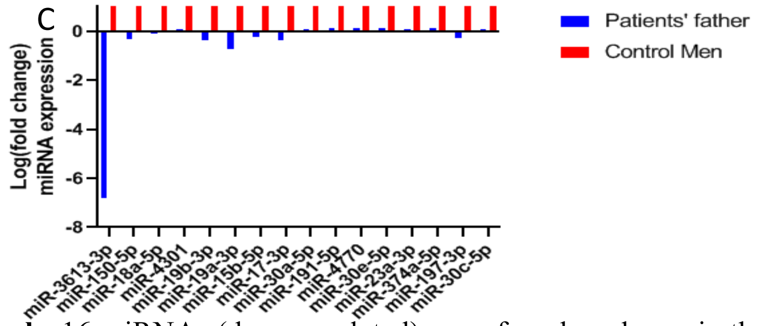

Fig.1b Patients' fathers compared to healthy male controls. 16 miRNAs (downregulated) were found as shown in the graph (C) and table (D).

D

miRNA	Log(fold change) Patients' fathers (n=37)	Log(fold change) Control Men (n=8)	miRNA	Log(fold change) Patients' fathers (n=37)	Log(fold change) Control Men (n=8)
miR-3613-3p	-6.80606	1	miR-30a-5p	-0.03665	1
miR-150-5p	-0.31346	1	miR-191-5p	-0.02524	1
miR-18a-5p	-0.09276	1	miR-4770	-0.01006	1
miR-4301	-0.04901	1	miR-30e-5p	-0.03094	1
miR-19b-3p	-0.37706	1	miR-23a-3p	-0.04609	1
miR-19a-3p	-0.71662	1	miR-374a-5p	-0.02271	1
miR-15b-5p	-0.25856	1	miR-197-3p	-0.26137	1
miR-17-3p	-0.3618	1	miR-30c-5p	-0.05194	1

E

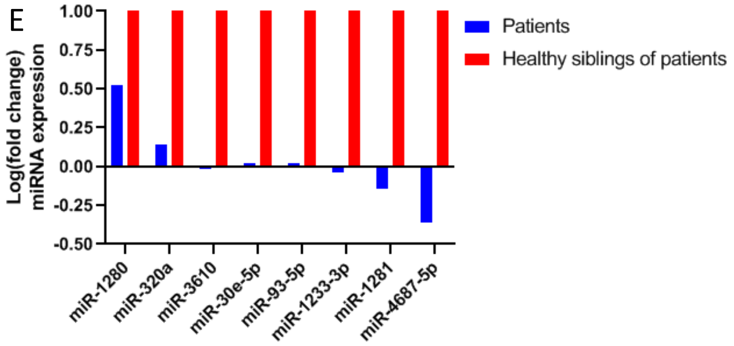

Fig.1c miRNA Autism patients compared to their healthy siblings. 8 miRNAs (3 up- and 5 downregulated) were found as shown in the graph (E) and table (F).

F

	miR-1280	miR-320a	miR-3610	miR-30e-5p	miR-93-5p	miR-1233-3p	miR-1281	miR-4687-5p
Log(fold change) Patients (n=45)	0.523376	0.141137	0.008134	-0.00237	-0.00445	-0.03715	-0.14263	-0.36016
Log(fold change) Healthy siblings of patients (n=33)	1	1	1	1	1	1	1	1

G

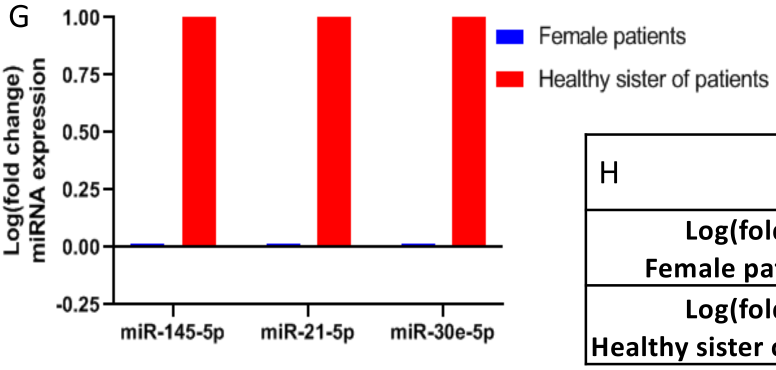

Fig.1d Female autism patients compared to the patients' sisters. 3 miRNAs (2 up- and 1 downregulated) were found as shown in the graph (G) and table (H).

H

	miR-145-5p	miR-21-5p	miR-30e-5p
Log(fold change) Female patients (n=14)	0.013661	0.013045	-0.00342
Log(fold change) Healthy sister of patients(n=16)	1	1	1

Groups	Number of common miRNAs	miRNAs
Fathers have more than one child with autism<br>Mothers have more than one child with autism<br>Patient has an sibling with autism	6	**miR-19a-3p (downregulated)**<br>**miR-361-5p (downregulated)**<br>**miR-3613-3p (downregulated)**<br>**miR-150-5p (downregulated)**<br>**miR-126-3p (downregulated)**<br>**miR-499a-5p (downregulated)**
Fathers have more than one child with autism<br>Patient has an sibling with autism	23	miR-363-3p, miR-17-3p, miR-32-5p, miR-1193 miR-18a-3p, let-7b-5p, miR-3159, miR-1280 miR-202-3p, miR-605-5p, miR-9-5p, miR-143-3p, miR-1307-3p, miR-421, miR-126-3p, miR-11111, miR-18a-5p, miR-576-5p, miR-660-5p, miR-151a-3p, miR-1301-3p, miR-4301 and miR-4732-5p
Mothers have more than one child with autism<br>Patient has an sibling with autism	16	miR-92a-3p, miR-130a-3p, miR-192-5p, miR-130b-3p, miR-16-5p, miR-19b-3p, miR-195-5p, miR-144-3p, miR-17-5p,NOTE miR-140-3p, miR-25-3p, miR-486-5p, miR-451a miR-425-5p, miR-30e-5p, miR-93-5p
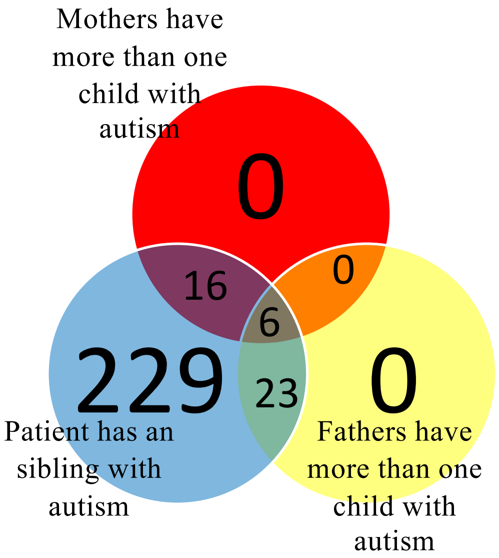 Patient has an sibling with autism	229	miR-629-5p, miR-942-5p, miR-181b-5p, miR-373-5p, miR-30a-3p, miR-495-3p, miR-4770 miR-548d-5p, miR-631 miR-337-3p, miR-1281 miR-433-3p, miR-34a-5p, miR-100-5p, miR-223-5p, miR-324-5p, miR-542-3p, miR-671-3p, miR-10a-5p, miR-1 miR-424-5p, miR-574-3p, miR-30e-3p, miR-378a-5p, miR-486-3p, miR-7-5p, miR-128-3p, miR-374b-5p,NOTE miR-4286 miR-103a-3p, miR-320b miR-339-3p, miR-184 miR-28-3p, miR-190a-5p, miR-26a-5p, miR-769-5p, miR-503-5p, miR-627-5p, miR-320e miR-183-5p, miR-422a miR-134-5p, miR-222-3p, miR-3911 miR-106b-5p, miR-122-5p, miR-378g miR-324-3p, miR-744-5p, let-7i-5p, miR-3689a-5p,NOTE miR-423-3p, miR-375 miR-487a-3p, miR-181a-5p, miR-454-3p, miR-3646 miR-378h miR-29b-3p, miR-191-5p, miR-497-5p, miR-183-3p, miR-382-5p, miR-21-5p, miR-28-5p, miR-133b miR-4524a-3p, miR-15b-3p, miR-374a-5p, miR-181d-5p, miR-362-3p, miR-27a-3p, miR-101-3p, miR-1260a miR-4651 miR-152-3p, miR-3183 miR-329-3p, miR-125a-5p, miR-425-3p, miR-1180-3p, miR-4323 miR-1260b miR-615-5p, let-7e-5p, miR-151bNOTE miR-139-5p, miR-7-2-3p, miR-214-3p, miR-625-3p, miR-342-5p, miR-339-5p, miR-487b-3p, miR-550a-5p, miR-16-2-3p, miR-145-5p, miR-2110 miR-1976 miR-345-5p, miR-1207-5p, miR-146b-5p, miR-628-3p, miR-27b-3p, miR-505-3p, miR-365a-3p,NOTE miR-224-5p, miR-4732-3p, miR-3651 miR-301a-3p, miR-145-3p, let-7c-5p, miR-3191-3p, let-7a-3p, miR-4274 miR-125b-5p, miR-22-5p, miR-4422 miR-1587 miR-489-3p, miR-3120-3p, let-7f-1-3p, miR-1910-5p, miR-30d-5p, miR-7-1-3p, miR-23a-3p, miR-376c-3p, miR-369-3p, miR-181c-3p, miR-3131 miR-15a-5p, miR-532-5p, miR-185-5p, miR-199a-5p, miR-29a-3p, miR-4454 miR-188-5p, miR-99b-5p, miR-3610 miR-98-5p, miR-26b-5p, miR-296-5p miR-664a-3p, miR-219a-1-3p, miR-378a-3p, miR-590-5p, miR-338-3p, miR-20a-5p, miR-381-3p, miR-1237-3p, miR-331-3p, miR-92b-3p, let-7g-5p, miR-142-3p, miR-373-3p, miR-1287-5p, miR-30a-5p, miR-186-5p, miR-197-3p, miR-29c-3p, miR-223-3p, miR-15b-5p, miR-4538 miR-30c-5p, miR-4306 miR-15a-3p, miR-20b-5p, miR-22-3p, miR-328-3p, miR-4296 miR-885-5p, miR-340-5p, miR-342-3p, miR-326 miR-191-3p, miR-3200-5p, miR-23b-3p, miR-490-3p, miR-126-5p, miR-409-3p, miR-4505 miR-637 miR-485-3p, miR-4687-5p, miR-4291 miR-148b-3p, miR-21-3p, miR-1913 miR-215-5p, miR-132-3p, miR-200c-3p, miR-378b miR-877-5p, miR-200b-3p, miR-1233-3p, miR-502-3p, miR-148a-3p, miR-99a-5p, let-7d-3p, miR-194-5p, miR-151a-5p, miR-142-5p, miR-3923 miR-378i miR-4516 miR-335-5p, miR-301b miR-144-5p, miR-766-3p, miR-596 miR-484 miR-3185 miR-24-3p, miR-675-3p, miR-130b-5p, miR-652-3p, miR-199b-5p, miR-30b-5p, miR-146a-5p, miR-877-3p, miR-1247-5p, miR-1225-3p, let-7f-5p, miR-181c-5p, miR-206 let-7a-5p, miR-199a-3p,NOTE miR-519b-5p,NOTE miR-370-3p,

According to a p value<0.001 in the microRNA analysis, six common the “Six-miRNAs” were identified.

Supplementary Figure 1I

Supplementary Figure 2

Agarose gel images of PCR products: *Cc2d1a* family genotyping.

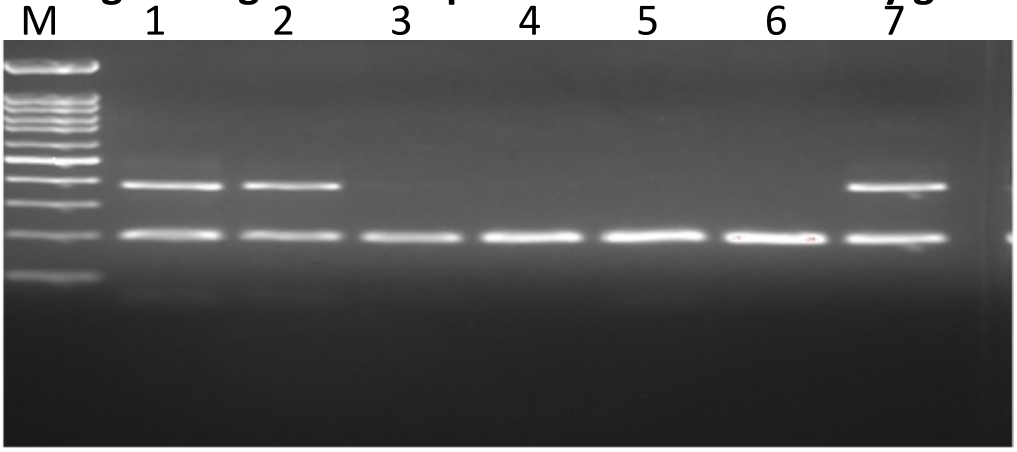

Supplementary Fig.2 Lines 1, 2 and 7 are heterozygous for *Cc2d1a* gene (see primer in Materials and Methods). Two fragments are produced (200 and 400 base pair (bp)), lines 3, 4, 5 and 6 wild type normal diploid genotypes with only 200 bp fragment.

Normal (200 bp), heterozygote (200 and 400 bp), M: Marker (100-1500 bp)

Supplementary Fig. 3 miRNAs analysis in mouse Blood and Hippocampus

Raw data are presented in a graph and table for the 5p strand (6) and the 3p premicroRNA (5) of the “Six-miRNAs” differentially expressed. (founder generation).

Supplementary Fig.3a samples from VPA-treated males 500 mg/ml (founder generation). Blood samples (A) graph (B) Table

Supplementary Fig.3b Hippocampus (C) graph (D) Table

A

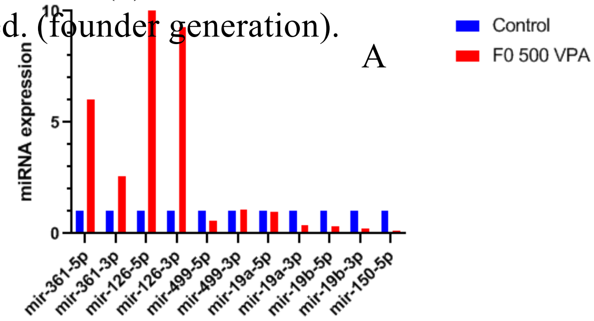

B

	Fold Change 500 mg/kg VPA (n=5)	Fold Change Control (n=5)
miR-361-5p	6.026	1
miR-361-3p	2.560	1
miR-126-5p	11.086	1
miR-126-3p	9.297	1
miR-499-5p	0.585	1
miR-499-3p	1.057	1
miR-19a-5p	0.984	1
miR-19a-3p	0.352	1
miR-19b-5p	0.308	1
miR-19b-3p	0.244	1
miR-150-5p	0.118	1

C

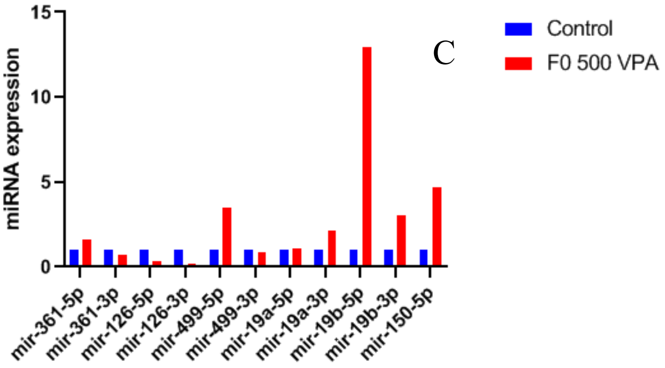

D

	Fold Change 500 mg/kg VPA (n=5)	Fold Change Control (n=5)
miR361-5p	1.61	1
miR361-3p	0.74	1
miR-126-5p	0.35	1
miR-126-3p	0.21	1
miR499-5p	3.49	1
miR499-3p	0.86	1
miR-19a-5p	1.06	1
miR-19a-3p	2.16	1
miR-19b-5p	12.94	1
miR-19b-3p	3.01	1
miR150-5p	4.67	1

E

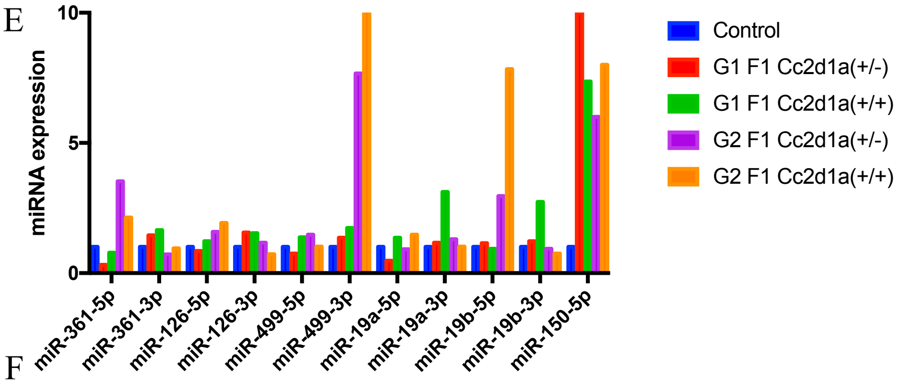

Supplementary Fig.3c Blood samples (E) graph (F) Table males from the *Cc2d1a* +/- family group compared to the control.

F

	miR--361-5p	miR-361-3p	miR--126-5p	miR--126-3p	miR-499-5p	miR-499-3p	miR--19a-5p	miR--19a-3p	miR--19b5p	miR--19b-3p	miR-150-5p
Control (n=5)	1.000	1.000	1.000	1.000	1.000	1.000	1.000	1.000	1.000	1.000	1.000
G1 F1 Cc2d1a(+/-) (n=5)	0.32	1.45	0.85	1.56	0.75	1.36	0.48	1.17	1.15	1.23	10.00
G1 F1 Cc2d1a(+/+) (n=5)	0.79	1.65	1.23	1.54	1.38	1.74	1.36	3.12	0.94	2.74	7.36
G2 F1 Cc2d1a(+/-) (n=5)	3.53	0.72	1.59	1.17	1.48	7.67	0.92	1.30	2.96	0.94	6.00
G2F1 Cc2d1a(+/+) (n=5)	2.14	0.95	1.93	0.73	1.02	10.00	1.48	1.02	7.84	0.75	8.00

G

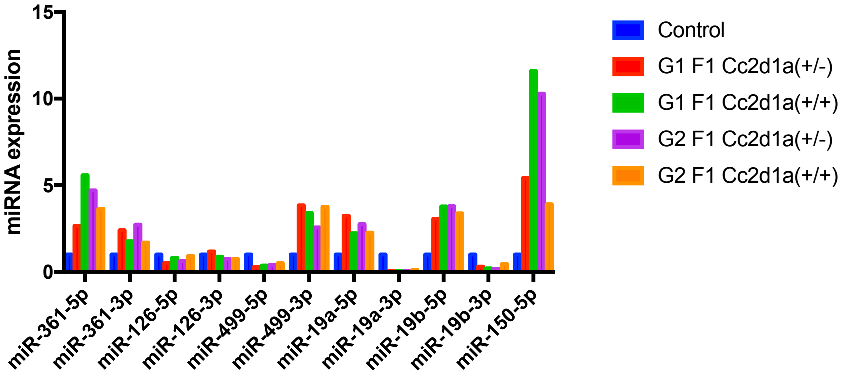

Supplementary Fig.3c Blood samples (G) graph (H) Table males from the *Cc2d1a* +/- family group compared to the control.

H

	miR--361-5p	miR-361-3p	miR--126-5p	miR--126-3p	miR-499-5p	miR-499-3p	miR--19a-5p	miR--19a-3p	miR--19b5p	miR--19b-3p	miR-150-5p
Control (n=5)	1.000	1.000	1.000	1.000	1.000	1.000	1.000	1.000	1.000	1.000	1.000
G1 F1 Cc2d1a(+/-) (n=5)	2.66	2.41	0.53	1.18	0.29	3.84	3.24	0.05	3.07	0.31	5.42
G1 F1 Cc2d1a(+/+) (n=5)	5.58	1.77	0.82	0.88	0.37	3.41	2.23	0.05	3.78	0.20	11.59
G2 F1 Cc2d1a(+/-) (n=5)	4.70	2.73	0.62	0.75	0.41	2.57	2.76	0.05	3.80	0.18	10.29
G2F1 Cc2d1a(+/+) (n=5)	3.64	1.69	0.92	0.74	0.50	3.76	2.27	0.12	3.38	0.45	3.91

Supplementary Fig. 4
Pie chart reflecting the relative percentages of newly identified miRNA target genes.
Predicted miRNA target genes in (**a**), *Mus musculus*, including miR-19a-3p, miR-361-5p, miR-150-5p, miR-126-3p, and miR-499a-5p and (**b**) *Homo sapiens*, including miR-19a-3p, miR-361-5p, miR-3613-3p, miR-150-5p, miR-126-3p, and miR-499a-5p. Mouse target genes for miR-3613-3p are unknown. Each group of predicted target genes of these miRNAs with conserved sites was selected from the most recently updated list from TargetScan7.2.

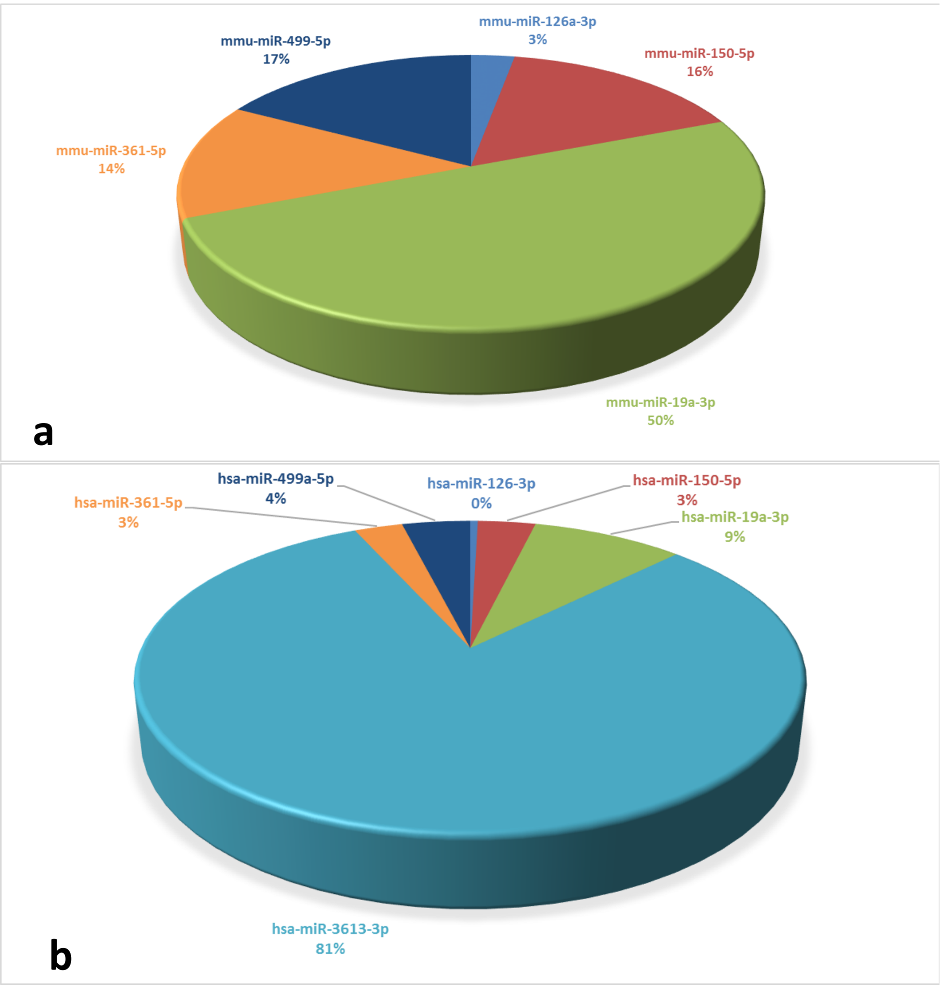

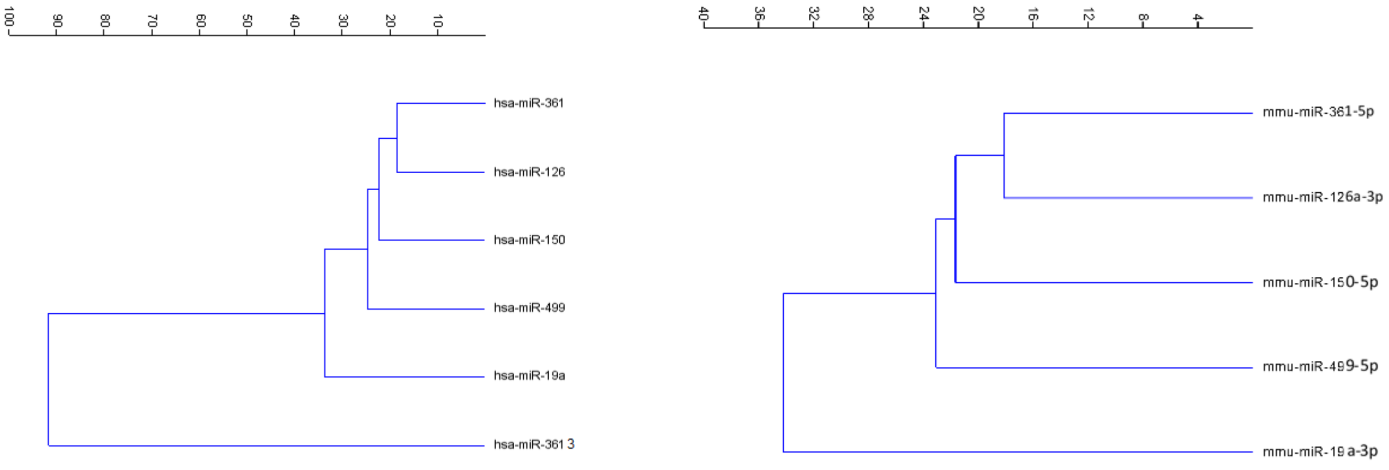

Supplementary Fig. 4c Hierarchical clustering dendrogram displaying the similarity between the genes targeted by each of six *Homo sapiens* miRNAs: miR-19a-3p, miR-361-5p, miR-3613-3p, miR-150-5p, miR-126-3p, and miR-499a-5p (left), and each of five *Mus musculus* predicted target microRNAs: miR-19a-3p, miR-361-5p, miR-150-5p, miR-126-3p, and miR-499a-5p (right). For both groups, the distinction between the miRNAs is based on the suggested target genes. The horizontal axis represents the distance between miRNA clusters. This demonstrates that each group of miRNAs in one cluster exhibits more common targeted genes or is very close together. As an example, mmu-miR-361-5p and mmu-miR-126-3p show more similarity, while hsa-miR-3613 appears to present more target genes than any of the other microRNAs. Predicted target genes with conserved sites were downloaded from TargetScan7.2.

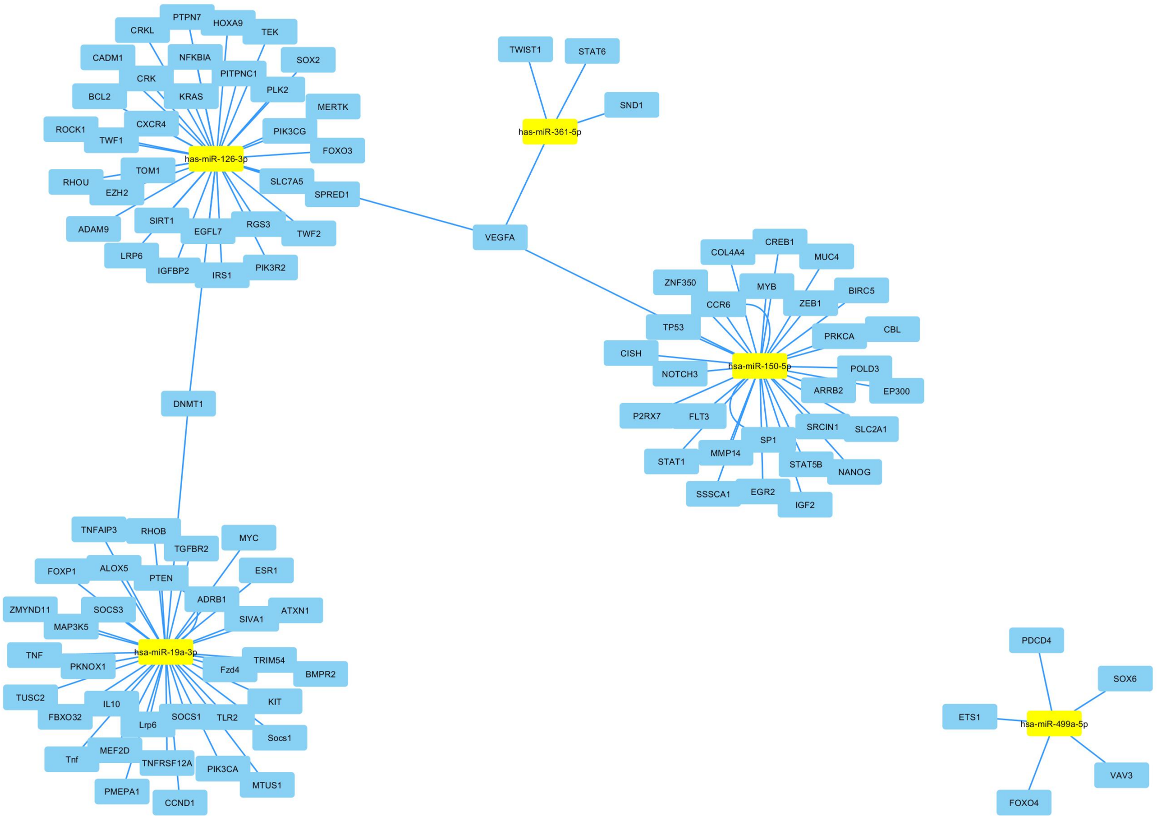

Supplementary Fig. 4d- Visualization of miRNA-target interaction using miRTarBase network where yellow squares refer to miRNAs, while blue ones refer to their target genes. With this program, for five out of six miRs multiples target genes are found, but any target for miR-3613-3p.

Supplementary Table 2 The six miRNAs sequences.
Sequences of the "Six-microRNAs" which was found downregulated in all autistic children

**miRNAs**	**Sequence**
miR-19a-3p	GUGCAAAUCUAUGCAAAACUGA
miR-361-5p	UUAUCAGAAUCUCCAGGGGUAC
miR-3613-3p	UGUUGUACUUUUUUUUUUGUUC
miR-150-5p	UCUCCCAACCCUUGUACCAGUG
miR-126-3p	UCGUACCGUGAGUAAUAAUGCG
miR-499a-5p	UUAAGACUUGCAGUGAUGUUU

Supplementary Table 3 Numbers of animals under study after valproic acid treatment (founder F0) and their progenies (F: Female, M: Male). At 14 days post birth, all offspring (8-12 pups from each litter) were injected (intraperitoneally) with sodium salt VPA at 300 to 700 mg/ml or with saline buffer as a control. Only 10 males were used for the behavioral tests and 5 for the miRNA evaluation.

Strain	Generation	Saline		300 mg/kg		400 mg/kg		500 mg/kg		600 mg/kg		700 mg/kg	
		F	M	F	M	F	M	F	M	F	M	F	M
B6/D2	F0	4	10	6	10	2	9	5	8		1	2	2
	F1					6	6	16	13				
	F2							12	14				
Balb/c	F0	7	9	14	11	11	11		6				
	F1			9	10	21	12	9	9				
	F2			6	14	12		10	10				
	F0	7	9	6	3	3	3		2				
	F1			5	5	6	11	9	9				
	F2												

Supplementary Table 4 Samples and list of miRNAs Samples and sequences of the microRNAs used in the mouse models.

Group Name	Behavioural Test	miRNA expression samples	Names of miRNAs	Sequences of miRNAs
F0 300 VPA	10 males from each group<br>Novel object<br>Social interaction<br>Tail suspension<br>Marble burying	5 males from each group<br>Sperm<br>Blood<br>Hippocampus	mmu-miR-150-5p	UCUCCCAACCCUUGUACCAGUG
F0 400 VPA			mmu-miR-150-3p	CUGGUACAGGCCUGGGGGAUAG
F0 500 VPA			mmu-miR-126a-3p	UCGUACCGUGAGUAAUAAUGCG
F1 500 VPA			mmu-miR-126a-5p	CAUUAUUACUUUUGGUACGCG
Saline Control			mmu-miR-361-5p	UUAUCAGAAUCUCCAGGGGUAC
Control			mmu-miR-361-3p	UCCCCCAGGUGUGAUUCUGAUUUGU
Group1 *Cc2d1a(+/+)*			mmu-miR-499-5p	UUAAGACUUGCAGUGAUGUUU
Group1 *Cc2d1a (+/-)*			mmu-miR-499-3p	GAACAUCACAGCAAGUCUGUGCU
Group2 *Cc2d1a(+/+)*			mmu-miR-19a-3p	UGUGCAAAUCUAUGCAAAACUGA
Group2 *Cc2d1a(+/-)*			mmu-miR-19a-5p	UAGUUUUGCAUAGUUGCACUAC
Human Sperm (Father of two patients)	1 man	Sperm	mmu-miR-19b-3p	UGUGCAAAUCCAUGCAAAACUGA
Human Sperm Control	3 men	Sperm	mmu-miR-19b-1-5p	AGUUUUGCAGGUUUGCAUCCAGC

Supplementary Table 5 miRNAs gene target

Some of the candidate genes indicated to be involved in autism by in silico analysis share common microRNAs that target their transcripts and are mostly expressed at the early stage of development.

Gene	microRNA		Est in early embryos	
	Mouse	Human	Mouse	Human
Nlgn1		miR-495	+	+
Pten	miR-495<br>miR-19a<br>miR-19b	miR-495<br>miR-19a	+	+
Gabrb3		miR-499	-	+
Slc6a4			-	-
Fmr1	miR-495<br>miR-19a<br>miR-19b<br>miR-499	miR-19a<br>miR-19b<br>miR-499	+	+
Foxp2	miR-19a<br>miR-19b<br>miR-499	miR-19a<br>miR-19b<br>miR-499	-	-
Cadps2	miR-495	miR-495<br>miR-499		+
Cc2d1a	miR-19a<br>miR-19b		+	+
